# Highly Sensitive and Stable CeLaCuO/Ni-BTC MOF-Based Humidity Sensor for Plant Monitoring

**DOI:** 10.1021/acsami.5c16966

**Published:** 2025-12-06

**Authors:** Jolina Rodrigues, Karthik Chimatahalli Santhakumar, Hamid Nawaz, Swati Singh, Smruti Medha Mishra, Dalaver Hussain Anjum, Kyriaki Polychronopoulou, Nouha Alcheikh

**Affiliations:** 1 Department of Mechanical and Nuclear Engineering, 105955 Khalifa University of Science and Technology, Main Campus, Abu Dhabi, P.O. Box 127788, UAE; 2 Center for Catalysis and Separations (CeCaS), Khalifa University of Science and Technology, Main Campus, Abu Dhabi, P.O. Box 127788, UAE; 3 Department of Physics, 105955 Khalifa University of Science and Technology, 127788 Abu Dhabi, United Arab Emirates

**Keywords:** humidity sensing, CeLaCuO/Ni-BTC composite, ternary metal oxide, MOF-based sensors, cerium oxide, plant health monitoring

## Abstract

Herein, we report a high-performance humidity sensor based on oxygen-vacancy-rich CeLaCuO integrated with a porous Ni-BTC metal–organic framework (MOF). Compared with the single CeLaCuO and Ni-BTC sensors, the CeLaCuO/Ni-BTC composite sensor exhibits a higher response value (24% @ 32% relative humidity (RH)), lower hysteresis (0.465%RH), faster response/recovery time (24.5/47.8 s), and enhanced long-term stability (<2.6% over 60 days). Moreover, it achieves a high sensitivity of *S* ≈ 1.35/%RH with excellent linearity (*R* ^2^ = 0.9868) across 11–63% RH and demonstrates a very low temperature cross-sensitivity between 25 and 100 °C (<0.35%). These improved performance properties are attributed to abundant oxygen vacancies (Ov) in the CeLaCuO structure that provide active sites for water adsorption and H^+^/H_3_O^+^ species generation for fast ionic conduction. The high-surface-area Ni-BTC framework enhances water uptake and facilitates efficient charge transfer at the oxide–MOF interface. The lab-fabricated composite sensor also demonstrates real-world applicability in a microclimate chamber for monitoring the microclimate surrounding the *Fragaria ananassa* (strawberry) plant, where lower humidity (<60%) can cause plant stress and reduce yield. The proposed sensor placed on the plant shows a good response for various humidity levels at 43%, 51%, and 63% RHs, respectively. Moreover, the results show that, at 63% RH, plants exhibited optimal transpiration, allowing efficient water and nutrient uptake, resulting in healthy leaf morphology with minimal stress. Thus, the proposed sensors hold strong potential as next-generation real-time humidity sensors with practical applications in agriculture, smart greenhouses, environmental monitoring, and indoor climate control.

## Graphical abstract

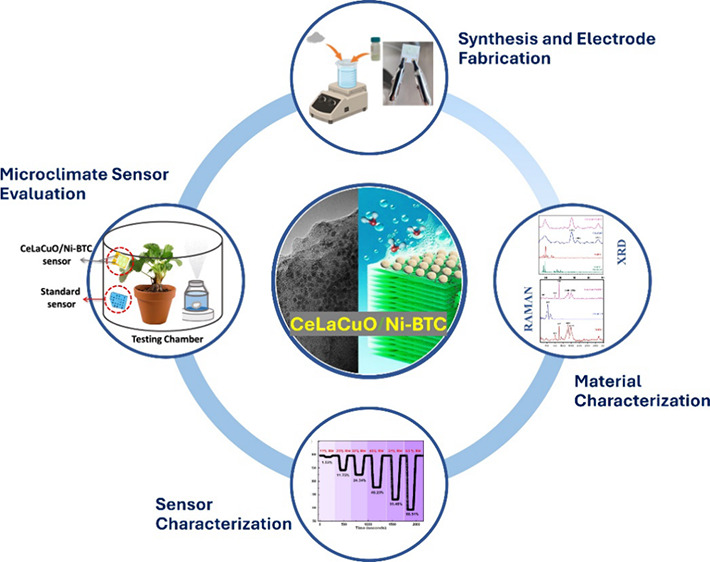

## 1 Introduction

Humidity sensors are critical in various industrial sectors such as healthcare, agriculture, environmental monitoring, semiconductor manufacturing, and textiles, where precise humidity control enhances process efficiency and product quality.^1,2^ In environmental monitoring, humidity sensors measure and regulate humidity levels accurately, maintaining optimal environmental conditions. The market for humidity sensors is expanding, driven by the growing demand for smart, flexible, and efficient sensing devices that can be integrated into wearable electronics, industrial automation, and next-generation environmental monitoring systems.^3,4^ As research advances, various humidity sensing technologies have been developed, including electrical signal-based sensors, optical fiber sensors, field-effect transistor (FET) sensors, surface acoustic wave (SAW) sensors, and quartz crystal microbalance (QCM) sensors.^1,5^ These technologies offer unique advantages in sensitivity, response time, and integration capabilities.^6^ However, challenges remain in achieving high-performance humidity sensors that combine rapid response/recovery times, long-term stability, selectivity, and low power consumption for diverse applications.^2,7^

Among emerging materials, metal–organic frameworks (MOFs) have garnered significant attention for gas and humidity sensing due to their high porosity, tunable chemical functionality and structure, and selective adsorption properties.^8,9^ MOF-based capacitive sensors have demonstrated high sensitivity, long-term stability, and low power consumption, making them suitable for next-generation sensor technologies. For instance, Gao et al. developed MIL-101­(Cr)-based photonic vapor sensors and fabricated optical MOF sensors responsive to various organic vapors.^10^ Similarly, Wang et al. reported a ZIF-8-based optical CO_2_ sensor and developed a capacitive Mg-MOF-74 sensor for selective benzene and CO_2_ detection.^11^ These works highlight MOF’s potential in humidity and gas sensing, particularly in applications requiring fast, selective, and energy-efficient detection.^12–14^

In addition to MOFs, metal-oxide hybrids, particularly those derived from MOFs, are widely explored for humidity sensing due to their high mechanical strength, thermal stability, and rapid response times.^2,15^ Oxygen vacancies play a crucial role in enhancing the sensing performance of these materials by facilitating charge transfer and increasing the density of reactive adsorption sites.^16^ However, a significant challenge in humidity sensing is the structural decomposition of Cu-based MOFs, such as Cu-BTC, under prolonged humidity exposure, leading to crystallinity loss and reduced adsorption capabilities.^17,18^ To address this, approaches such as Fe-BTC nanoparticle incorporation, core–shell hybridization with copper phosphate nanoflowers, and in situ polymer functionalization have been explored to enhance water resistance and maintain sensor performance.^19^ Unfortunately, hydrophobic polymer coatings, while improving water resistance, can reduce pore accessibility and thus potentially affect the MOF’s overall sensing efficiency. Hence, balancing structural stability and functional performance under humid conditions remains a key design challenge.^20^

Our overarching strategy is to incorporate the oxygen vacancy (Ov) rich hydrophilic ternary metal oxide (CeLaCuO) into the Ni-BTC structure so to synergistically enhance the humidity sensing performance.^21^ By hybridizing CeLaCuO with Ni-BTC, a hierarchical material is expected to exhibit enhanced water adsorption behavior due to the presence of oxygen vacancies and the porosity of MOFs, which increase the material’s hydrophilicity and active sites for water molecule interactions.^22^ The rich population of Ov in the CeLaCuO structure has been demonstrated in previous reports by our group.^23^ When combined with MOFs, which are renowned for their high porosity and tunable frameworks, the resulting hybrid material can offer a synergistic effect that both enhances structural robustness and promotes favorable interactions with water molecules under humid conditions.^24^ MOF structures, like Ni-BTC, are anticipated to provide a large specific surface area (m^2^/g) and tunable pore environments, which are advantageous for humidity sensing as they facilitate rapid diffusion pathways and maintain structural integrity under varying humidity (11–64%RH).^24^ The hierarchical structure formed by hybridizing CeLaCuO with Ni-BTC as shown in Figure 1a can thus leverage the strengths of both components: the high-water uptake and stability of MOFs and the enhanced sensitivity and rapid response of metal oxides with oxygen vacancies.^25^

**Figure 1:**
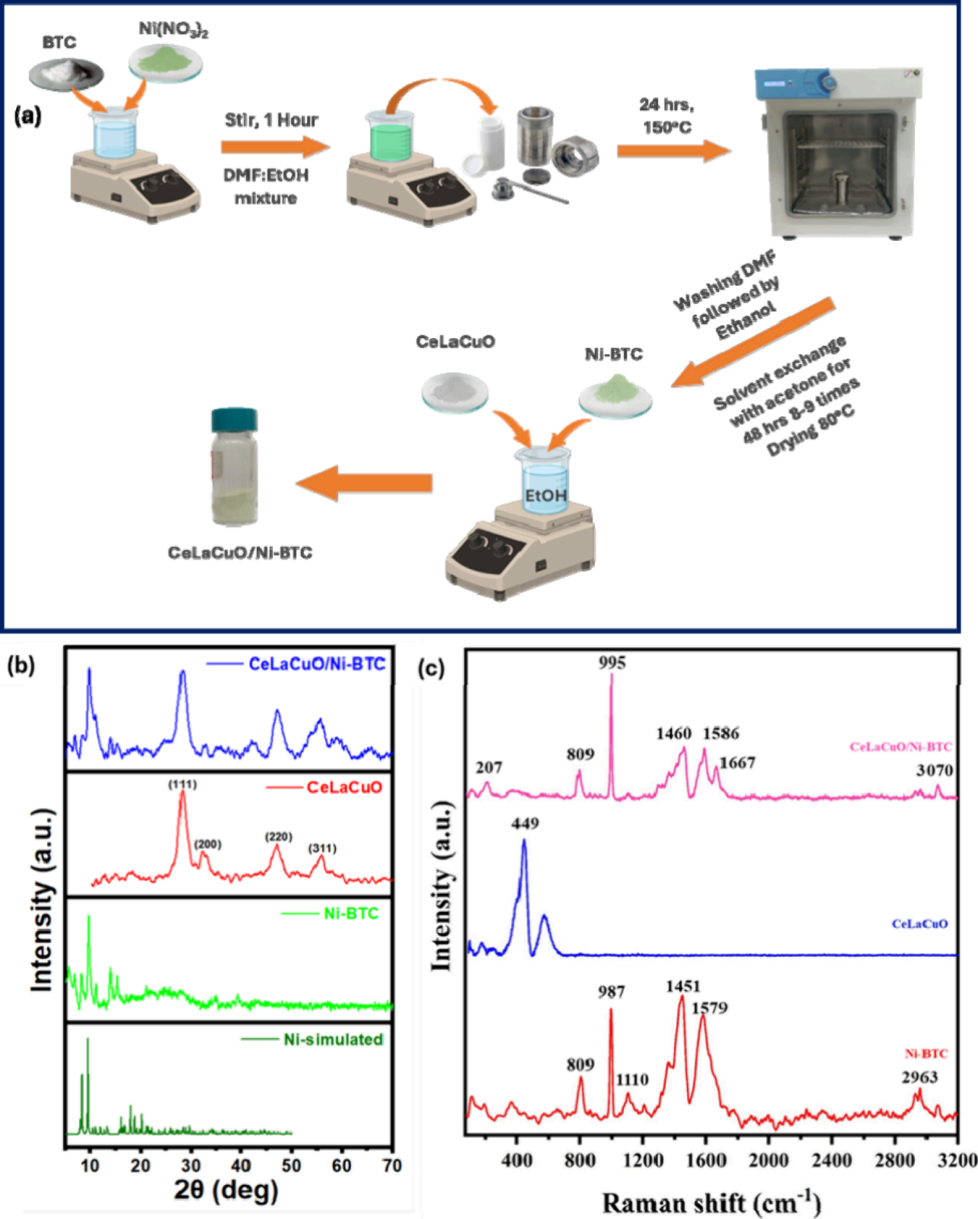
(a) Schematic representation of the synthesis route for the CeLaCuO/Ni-BTC composite. (b) XRD patterns of CeLaCuO, Ni-BTC, and their composite showing crystallinity and phase composition. (c) Raman spectra of CeLaCuO, Ni-BTC, and CeLaCuO/Ni-BTC composite.

In this context, we present a CeLaCuO/Ni-BTC MOF composite sensor where the hydrophilicity is primarily driven by the oxygen-vacancy-rich CeLaCuO oxide, which provides abundant active sites for water molecule adsorption and dissociation. Ni-based MOFs show promise for humidity sensing due to their tunable, porous structures that enhance water adsorption and provide active sites for detection.^26^

Integrating attributes from CeLaCuO (oxide structure), such as oxygen vacancies, enhances water molecule adsorption. In contrast, the ones coming from the Ni-BTC MOF porous framework ensure efficient moisture diffusion and adsorption dynamics.^27^

Furthermore, by leveraging the synergistic characteristics of both components, this composite overcomes the limitations in response/recovery time (24.5/47.8 s), excellent long-term stability (60 days), high sensitivity (*S* ≈ 1.35/%RH), and a very low temperature cross-sensitivity between 25 and 100 °C, providing a promising platform for next-generation humidity sensors. Moreover, the proposed sensor proved its real-world utility by monitoring the microclimate around a *Fragaria ananassa* (strawberry) plant in a controlled chamber. The sensor response revealed that at 63% RH the plant achieved optimal transpiration, enhanced water and nutrient uptake, and maintained healthy leaf morphology with minimal stress. Thus, the proposed sensors hold strong potential as next-generation real-time humidity monitoring tools, offering reliable and precise environmental feedback. Their proven stability, sensitivity, and responsiveness make them well-suited for diverse practical applications, including precision agriculture, smart greenhouse systems, environmental monitoring, and indoor climate regulation, where maintaining optimal humidity levels is crucial for health, productivity, and sustainability.

## 2 Materials and Methods

### 2.1 Materials

All reagents were of analytical grade and used as received without further purification. *N*,*N*-Dimethylformamide (DMF) was obtained from Fischer Chemicals, while benzene-1,3,5-tricarboxylic acid (H_3_BTC), nickel nitrate hexahydrate (Ni­(NO_3_)_2_·6H_2_O, 97.0%), cerium­(III) nitrate hexahydrate (Ce­(NO_3_)_3_·6H_2_O, 99.95%), lanthanum­(III) nitrate hexahydrate (La­(NO_3_)_3_·6H_2_O, 99.95%), and copper­(II) nitrate trihydrate (Cu­(NO_3_)_2_·3H_2_O, 99.95%) were obtained from Sigma-Aldrich (USA).

### 2.2 Synthesis of Ni-BTC-MOF

Nickel­(II) nitrate hexahydrate (1.75 g) and benzene-1,3,5-tricarboxylic acid (0.84 g) were dissolved in 50 mL of a mixed solvent system (*N*,*N*-dimethylformamide (DMF):ethanol, 1:1) under sonication for 30 min. After being stirred for 15 min, the solution was heated in an oven at 120 °C for 48 h. After being cooled to room temperature, the product was washed twice with DMF and methanol and then dried in a vacuum oven at 80 °C overnight to obtain Ni-BTC powder. The dried Ni-BTC MOF underwent a solvent exchange process using acetone for 3 days, with 4–5 wash cycles during this period.

### 2.3 Synthesis of CeLaCuO

CeLaCuO ternary oxide incorporating 10 at. % Cu content was prepared via a microwave assisted protocol with slight modifications from our previously reported protocol.^21^ To get a 45 at. % content of Ce and La, each metal precursor Ce­(NO_3_)_3_·6H_2_O, La­(NO_3_)_3_·6H_2_O, and Cu­(NO_3_)_2_·3H_2_O was dissolved in distilled water using the proper molar ratio. Both solid synthesis solutions maintained a total metal loading of 0.03 mol. Ethylene glycol was used as a complexing agent and dissolved in distilled water at a volume ratio of 2:1 (v/v, ethylene glycol:water). After mixing, the metal nitrate solution was then combined with ethylene glycol, and they were heated in a microwave at 170 °C while being constantly stirred. After centrifuging the mixture, the solid that was produced was calcined for 6 h at 500 °C in air.

### 2.4 Synthesis of the CeLaCuO/Ni-BTC Composite

To prepare the CeLaCuO/Ni-BTC composite, 10 wt % synthesized CeLaCuO nanoparticles were dispersed in ethanol and added to a presynthesized Ni-BTC MOF suspension in the same solvent. The resulting mixture was magnetically stirred continuously for 12 h at room temperature to ensure uniform distribution and interaction between the metal oxide and MOF components. After stirring, the composite material was separated by filtration, thoroughly washed with methanol to remove any residual impurities, and subsequently dried in an oven at 90 °C for 12 h.

### 2.5 Characterization

XRD analysis was carried out using an Anton Paar Panalytical diffractometer equipped with a Cu anode (*K*_α_1__ = 1.54060 Å, *K*_α_2__ = 1.54443 Å, and *K*_β_ = 1.39225 Å). The samples were analyzed with a diffractometer at a voltage of 45 kV and intensity of 40 mA. X-ray diffractograms were collected in the 2θ range of 5–50°, with a step size of 0.013°, and a scan speed of 40 steps/s. Raman spectroscopy was carried out to complement powder XRD and investigate the oxygen sublattice and induced structural defects. Measurements were performed using a WITec alpha300 RA+ confocal Raman microscope equipped with a 532 nm green laser as the excitation source. A 10× objective lens was used. Surface elemental compositions and chemical states were determined using X-ray photoelectron spectroscopy (XPS) on a ThermoScientific ESCALAB QXi spectrometer equipped with a monochromatic Al K_α_ X-ray source (photon energy ∼1486 eV). Transmission electron microscopy (TEM) analysis was performed using a Talos 200 (Thermo Fisher Scientific) operated at 200 kV. Bright-field TEM was employed to examine the sample morphology, while high-resolution TEM (HR-TEM) provided detailed structure visualization. Selected area electron diffraction (SAED) was used for crystal structure determination, and dark-field scanning transmission electron microscopy (DF-STEM) was utilized to image the morphology in dark-field mode. Energy-dispersive X-ray spectroscopy (EDS) enabled composition determination, and STEM-EDS was performed for elemental mapping to understand the spatial distribution of elements within the sample. The entire TEM data acquisition process was carried out by using Velox V 2.5 software.

## 3 Results and Discussion

### 3.1 Structural and Textural Characterization

The structural and compositional analysis of the synthesized Ni-BTC, CeLaCuO, and CeLaCuO/Ni-BTC composite was examined using powder XRD, as presented in Figure 1b. The XRD patterns of the Ni-BTC MOF, presented in Figure 1b, exhibit well-defined diffraction peaks, confirming the high crystallinity of the synthesized material. The observed diffraction pattern corresponds to the characteristic peaks of Ni-BTC MOF, which aligns well with previously reported literature.^31–33^ Additionally, the simulated diffraction pattern for Ni-BTC MOF was generated using single-crystal data from the Cambridge Crystallographic Data Centre (CCDC No. 985792), further validating the structural identity of the synthesized MOF. The sharpness of these peaks indicates the structural integrity and phase purity of the MOF.^30^

However, relative to the simulated Ni-BTC pattern, the presence of trace impurities cannot be entirely ruled out, and differences in crystallite size or preferred orientation may reasonably account for the observed slight variations in peak intensities. On the other hand, the diffraction peaks of the CeLaCuO/Ni-BTC composite observed at ∼28.5°, 33.1°, 47.4°, and 55.8° (2θ) correspond to the (111), (200), (220), and (311) crystal planes of the cubic fluorite structure of CeO_2_, in agreement with JCPDS 34–0394.^28,29^ This indicates that CeO_2_ remains as the primary phase in the CeLaCuO system. A noticeable peak shift toward lower diffraction angles was observed after the incorporation of La^3+^ and Cu^2+^ cations into the CeO_2_ lattice. This shift is attributed to the partial substitution of Ce^4+^ (0.97 Å) by larger La^3+^ (1.1 Å) and smaller Cu^2+^ (0.73 Å) cations. The variation in the ionic radii introduces lattice strain, altering the lattice parameters, which is indicative of homogeneous solid solution formation. The absence of distinct diffraction peaks corresponding to La_2_O_3_, CuO, or cerium–copper oxide heterophases suggests the effective incorporation of La and Cu ions into the CeO_2_ matrix. Importantly, the Ni-BTC reflections are retained in the CeLaCuO/Ni-BTC composite, confirming that the MOF framework remains intact upon integration with CeLaCuO. The broadening of peaks in the CeLaCuO/Ni-BTC MOF composite confirms partial crystallinity retention, while indicating the presence of structural defects and nanoscale effects. The incorporation of the oxygen vacancy (Ov) rich structure of CeLaCuO into the Ni-BTC MOF is expected to enhance the material’s adsorption and redox properties.^34^ The presence of these oxygen vacancies is crucial for humidity sensing applications, as they facilitate enhanced water molecule adsorption and charge transfer mechanisms.^35^

Raman spectroscopy was employed to investigate the vibrational properties and structural integrity of the CeLaCuO/Ni-BTC composite, providing insights into the chemical bonding, phase stability, and defect states. The Raman spectra of CeLaCuO, Ni-BTC composite, and their composites are presented in Figure 1c. The Raman spectrum of Ni-BTC MOF displays distinct peaks associated with the benzene-1,3,5-tricarboxylate (BTC) ligand and the Ni–O coordination framework. The presence of BTC ligand vibrations is evident from peaks at ∼809 cm^–1^ (aromatic C–H bending) and ∼987 cm^–1^ (benzene ring breathing mode), confirming the structural integrity of the MOF.^36^ Peaks at ∼1451 cm^–1^ and 1579 cm^–1^ correspond to symmetric and asymmetric carboxylate stretching, which validate the coordination of BTC ligands to Ni^2+^ ions.^36^ The Raman spectrum of CeLaCuO exhibits characteristic peaks associated with the fluorite CeO_2_ lattice, along with additional modes related to oxygen vacancies and Cu–O interactions. The prominent peak at ∼449 cm^–1^ corresponds to the F2g symmetric stretching mode of CeO_2_, confirming the retention of the fluorite structure.^37^ This band is slightly shifted from the typical 466 cm^–1^ peak of pure CeO_2_, indicating the incorporation of La^3+^ and Cu^2+^ cations into the ceria lattice, which induces lattice strain and oxygen vacancies.^37,38^ In the case of composite material, both CeLaCuO and Ni-BTC spectral features are retained, with a broadening of peaks in the 500–700 cm^–1^ range, suggesting strong interfacial interactions between the oxide and the MOF, facilitating charge transfer. A slight shift in the asymmetric carboxylate stretches (∼1586 cm^–1^ vs 1579 cm^–1^ in pure Ni-BTC) further indicates electronic interactions between Ni^2+^ and ceria centers and MOF ligands.^38^ Hence, the diminished oxygen vacancy in the composites is actively engaged in interfacial interactions, contributing to the composite functionality (charge transfer). These findings confirm that the CeLaCuO/Ni-BTC composite effectively integrates vacancy-rich metal oxides with porous MOF frameworks, making it a promising candidate for next-generation humidity sensors.

Nitrogen-sorption analysis and pore size distribution are presented in Figure 2a,b, confirming that all samples are mesoporous,^39^ with the CeLaCuO/Ni-BTC composite one showing the most favorable texture. As summarized in Table[Table tbl1], the parent Ni-MOF displays a BET surface area (SA_BET_) of 18.4 m^2^ g^–1^, a total pore volume (PV_total_) of 0.16 cm^3^ g^–1^, and an average pore diameter of 36 nm, consistent with open mesopores near the upper end of the IUPAC mesopore range (2–50 nm).^40^ The oxide CeLaCuO is weakly porous, with SA_BET_ = 9.83 m^2^ g^–1^, PV_total_ = 0.02 cm^3^ g^–1^, and pore size ≈ 9.8 nm. In contrast, the CeLaCuO/Ni-BTC composite exhibits a pronounced textural enhancement, reaching SA_BET_ = 32.5 m^2^ g^–1^ and PV_total_ = 0.30 cm^3^ g^–1^ while maintaining a mesopore diameter of ∼37 nm. The simultaneous increase in surface area and nearly 2-fold rise in pore volume, without narrowing of the mesopores, indicates that the oxide does not block MOF channels; instead, it contributes additional interfacial/interparticle mesoporosity, yielding a hierarchical mesoporous network that is advantageous for rapid adsorption and mass transport; both phenomena are of critical importance for the application of interest.^41–43^ The higher surface area and pore volume of the CeLaCuO/Ni-BTC composite compared with Ni-MOF are anticipated to increase the density of accessible adsorption sites for H_2_O at low–mid RH, thereby boosting humidity-sensor sensitivity.^44^ Its mesoporous average pore size (∼37 nm) is expected to provide low-resistance diffusion pathways, enabling faster response and recovery and helping suppress capillary-condensation-driven hysteresis, a known benefit of well-designed meso/macroporous networks.^45^ The larger total pore volume also supports the formation of continuous, hydrogen-bonded water films at high RH, which couple with defect/vacancy sites on the La/Cu-modified ceria and MOF channels to enhance protonic conduction via the Grotthuss mechanism, thereby increasing the conductivity change across RH and improving stability over temperature.^46^

**Figure 2:**
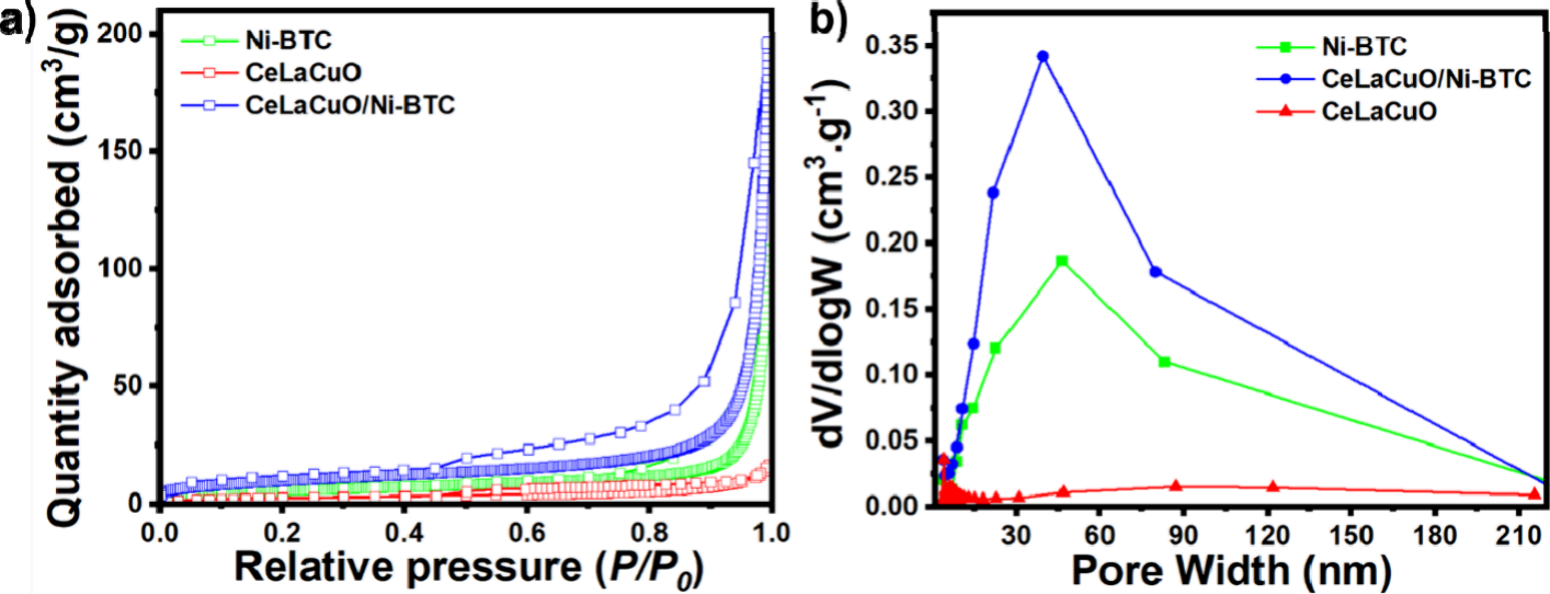
Physisorption characteristics of as-synthesized Ni-BTC, CeLaCuO, and CeLaCuO/Ni-BTC composite: (a) N_2_ adsorption–desorption isotherms and (b) the pore size distribution.

**Table 1:** Surface and Textural Properties of Ni-BTC, CeLaCuO, and the CeLaCuO/Ni-BTC Composite

catalyst	**surface area (SA**_**BET**_ **)** **(m** **^2^** **/g)**	**pore volume (P**V_**total**_ **)** **(cm** **^3^** **/g)**	pore size_av_ (nm)
Ni-BTC	18.4	0.16	36
LaCeCuO	9.83	0.02	9.8
CeLaCuO/Ni-BTC	32.5	0.30	37

### 3.2 Surface Chemistry and Oxygen Vacancies

X-ray photoelectron spectroscopy (XPS) as presented in Figure 3 was employed to analyze the surface elemental composition and chemical states with a focus on confirming oxygen-vacancy formation. The survey XPS spectrum of the CeLaCuO/Ni-BTC composite showed the presence of Ce, La, Cu, Ni, O, and C (from the organic linker), consistent with the material’s formulation. High-resolution XPS spectra provide deeper insight into oxidation states. The O 1s spectrum was deconvoluted into multiple components: a primary peak near ∼529.5 eV attributable to lattice oxygen (O_lattice_) in metal–oxygen bonds and a higher-binding-energy shoulder around ∼531.5–532 eV corresponding to adsorbed oxygen species associated with defect sites.

**Figure 3:**
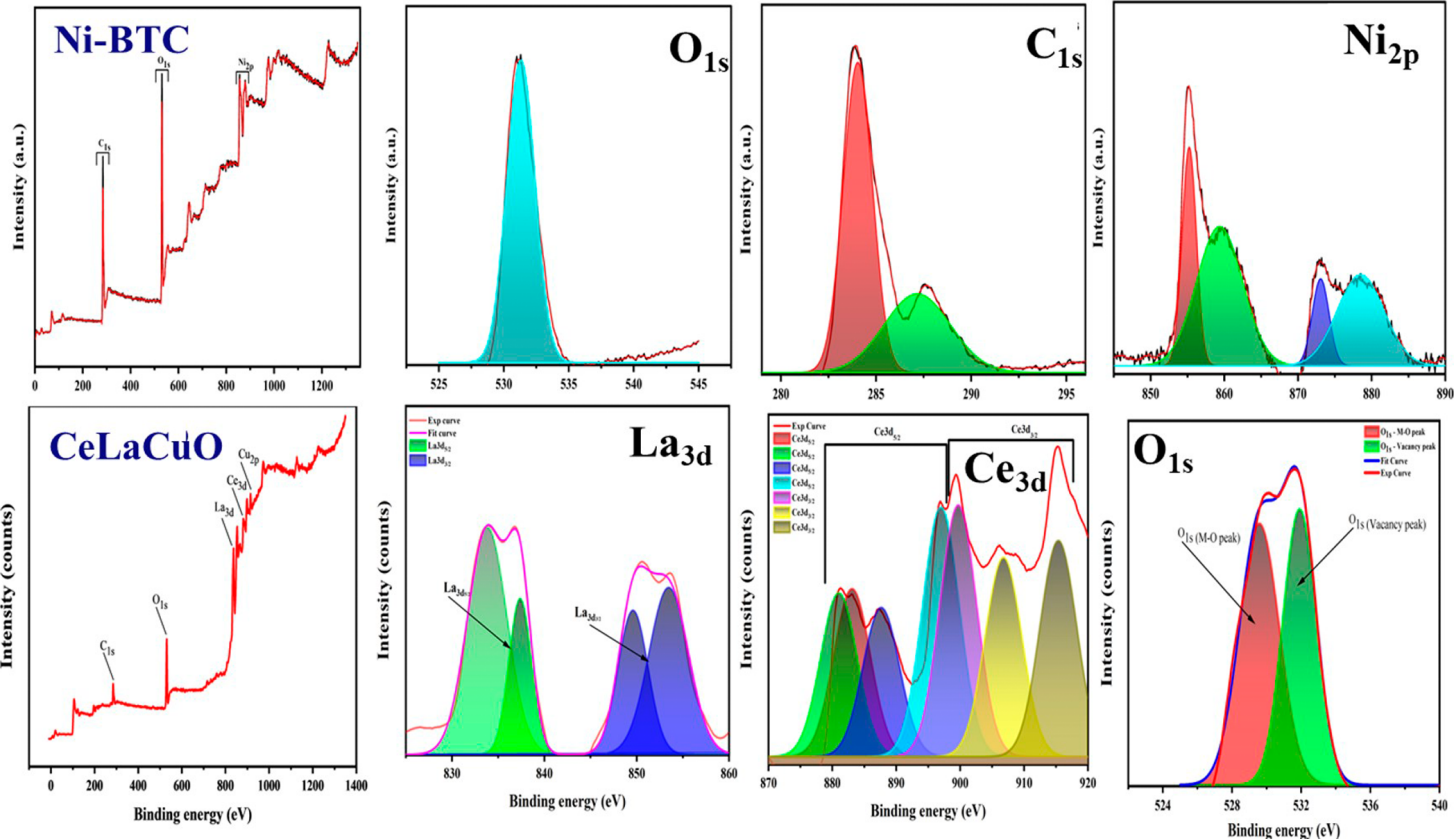
XPS spectra of CeLaCuO, Ni-BTC, and their composite.

This observation is in line with the Raman evidence of oxygen defects and confirms that doping with Ce and La introduced a high density of oxygen vacancy sites. It is well established that rare-earth dopants in metal oxides can generate additional oxygen vacancies, and our XPS results verify such defect engineering in CeLaCuO. The Ce 3d XPS spectrum further supports this conclusion. The Ce 3d region of the composite exhibits the characteristic set of spin–orbit-split peaks for Ce in mixed oxidation states. The presence of Ce^3+^ in the material directly implies oxygen-deficient cerium environments (Ce^3+^ is stabilized by oxygen vacancies in CeO_2_ based lattices). The Cu 2p spectrum indicates Cu^2+^ as the principal state (with Cu 2p_3/2_ ∼934 eV and its satellite features), while the Ni 2p spectrum confirms Ni^2+^ in the MOF nodes (Figure S1). The La 3d spectrum shows La^3+^ as expected. The key point is that the XPS data verify that our composite contains abundant oxygen vacancies (as reflected by the O 1s and Ce 3d signatures).

The inverse fast Fourier transform (IFFT) processed high-resolution TEM image provides enhanced visualization of the crystalline lattice structure within the CeLaCuO/Ni-BTC MOF composite (Figure S2). The clear lattice fringes indicate the structural integration of CeLaCuO nanoparticles within the porous Ni-BTC framework, signifying strong interfacial bonding between the two phases. The interplanar spacing observed in the image corresponds to the characteristic diffraction planes of CeO_2_ and the Ni-BTC framework, confirming the successful incorporation of CeLaCuO without disrupting the MOF crystallinity. Notably, slight lattice distortions in the CeLaCuO domain suggest the presence of oxygen vacancies, which are known to play a crucial role in enhancing humidity adsorption and charge transfer. The uniform lattice alignment at the interface between CeLaCuO and Ni-BTC further confirms that the composite maintains structural coherence, facilitating effective moisture diffusion and adsorption of the hybrid material. These findings validate the successful integration of CeLaCuO with Ni-BTC, reinforcing its potential for high-performance humidity sensing applications.

### 3.3 Morphology and Elemental Distribution

The structural morphology and elemental composition of the CeLaCuO/Ni-BTC composite were characterized using HRTEM and EDS elemental mapping, as depicted in Figure 4.^47,48^ The HRTEM image (Figure 4a–c) reveals a highly porous network with an ultrathin layered structure, characteristic of the Ni-BTC MOF.^48^ The presence of dark contrast regions indicates the successful incorporation of CeLaCuO nanoparticles within the MOF matrix. Notably, the well-defined crystalline domains confirm the retention of the structural integrity of Ni-BTC MOF posthybridization, ensuring a stable framework for enhanced performance.^49,50^ The hierarchical porosity observed in the composite is expected to facilitate efficient water molecule adsorption and diffusion, a key requirement for high-performance humidity sensing applications.^51^ The high-angle annular dark-field scanning transmission electron microscopy (HAADF-STEM) image (Figure S3) provides a contrast-enhanced visualization of atomic distribution, where bright regions correspond to CeLaCuO domains, indicating the presence of heavier atomic number elements (Ce, La, Cu) within the composite.^52^ The EDS elemental mapping (Figure 4c) further confirms the uniform distribution of Ce, La, Cu, Ni, C, and O, signifying the successful formation of a homogeneous hybrid structure without phase separation. The integration of CeLaCuO into the Ni-BTC MOF is further corroborated by the presence of oxygen vacancies, as evidenced by the oxygen distribution in EDS mapping.^53^ In Figure S1 and Figure S4, HR-TEM and HAADF-STEM images confirm the presence of nanocrystalline domains and uniform dispersion of metal cations, indicative of successful ternary oxide formation.

**Figure 4:**
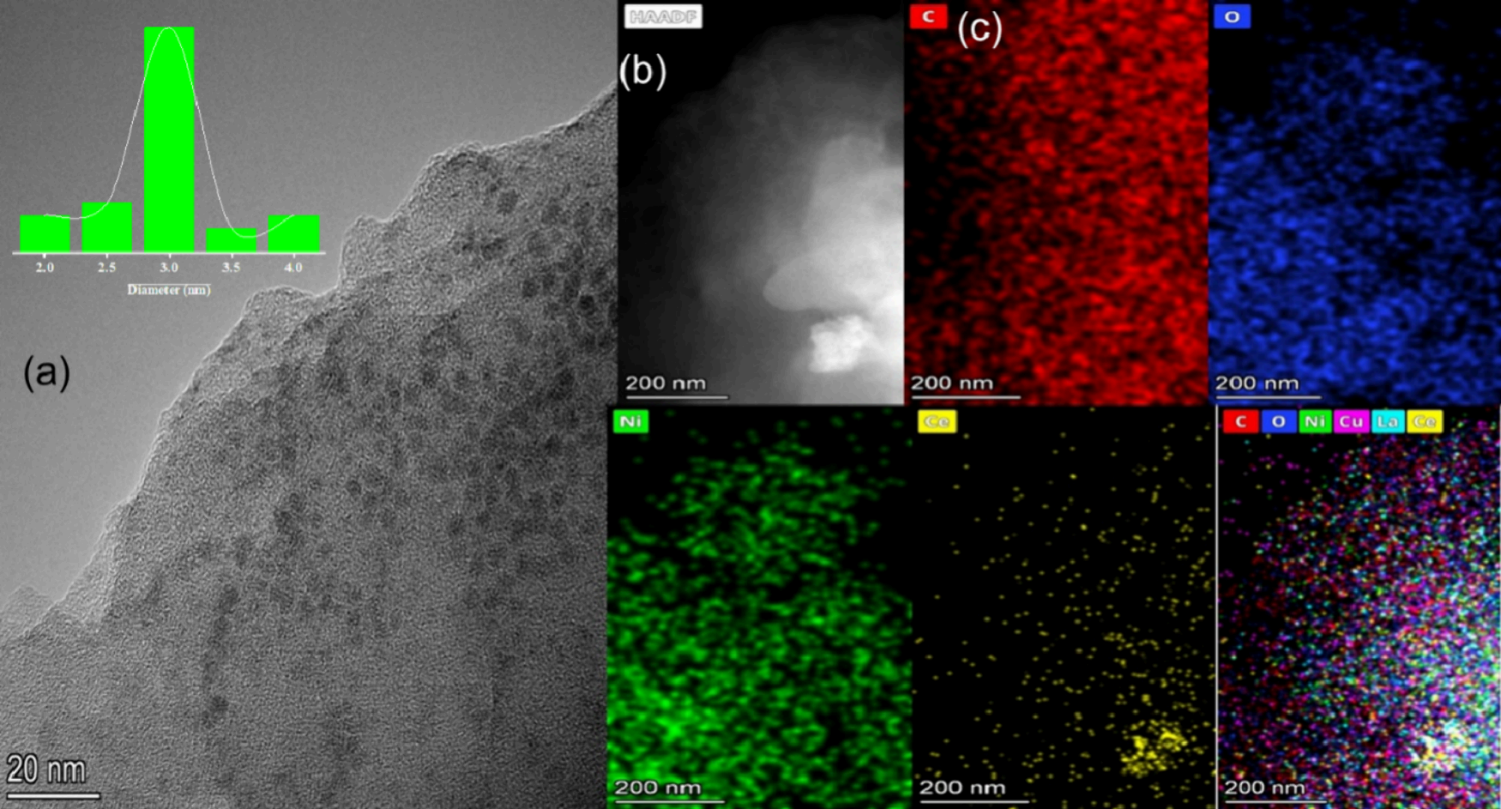
(a) HR-TEM of CeLaCuO/Ni-BTC MOF, showing the Ni-BTC MOF with incorporated CeLaCuO. Inset: particle size distribution histogram of CeLaCuO. (b) HAADF-STEM highlights atomic distribution. (c) EDS mapping confirms uniform element dispersion: Red - Carbon (C); Blue - Oxygen (O); Green - Nickel (Ni); and Yellow - Copper (Cu).

### 3.4 Humidity-Sensing Performance

A drop-casting technique was used to obtain the proposed sensors. A 10 mg sample of CeLaCuO/Ni-BTC composite was dispersed in 10 mL of ethanol and sonicated for 30 min to obtain a homogeneous suspension. Then, the solution was deposited onto interdigitate electrodes (IDEs), which were composed of Cr/Au with a line spacing of about 20 μm on a silicon wafer as a substrate. The coated IDE was dried at 70 °C for 30 min to evaporate residual ethanol prior to conducting humidity sensing measurements (Figure S5). Two independent sensors were fabricated under identical drop-casting conditions to assess the repeatability and device-to-device variation. The humidity responses (20–60% RH) showed consistent trends with minor sensitivity variations (±5%), confirming good reproducibility. The small deviation is attributed to slight differences in film thickness inherent to the drop-casting process (Figure S8). Thus, the humidity sensing performance of the composite CeLaCuO/Ni-BTC sensor compared to CeLaCuO and Ni-BTC sensors was tested under control conditions, where dry air (N_2_ gas) and humidified air were mixed using flow meters before entering a sealed test chamber. The reference commercial humidity sensor was placed in the microclimate chamber to ensure accurate control and calibration of the humidity levels during testing, as regulated by the flow meter. The purpose of this setup was to minimize experimental error and verify the stability of the humidity conditions within the chamber. The variation in the sensor’s electrical resistance was continuously recorded in real time using a digital multimeter (Keithley DMM 4050) connected to data acquisition software. The DMM offers a measurement precision of ±0.0020% of the reading plus ±0.0005% of the range (Figure S5). Figure 5a shows that the resistance response values of CeLaCuO, Ni-BTC, and CeLaCuO/Ni-BTC sensors at 11% RH are 3.8%, 4%, and 10.5%, respectively, demonstrating that the oxide MOF interface provided the structural attribute (porosity, functional groups, oxygen vacancy site) required for the investigated humidity sensing. The sensor response was calculated using (*R*_dry_ – *R*_humid_) × 100/*R*_dry_, where *R*_dry_ and *R*_humid_ represent the resistance value of the sensor in dry air and at a specific humidity level, respectively. Another test has been performed at 23%RH and 32% RH (Figure S6), proving that the composite material exhibits the highest response to humidity sensing, making it suitable for sensing low humidity. Moreover, the stability of the material toward temperature has been investigated to highlight the impact of temperature on the response shifts. As shown in Figure 5b, the results demonstrate the long-term stability of the sensors until 100 °C. Thus, the humidity CeLaCuO/Ni-BTC sensor was used for the following measurements.

**Figure 5:**
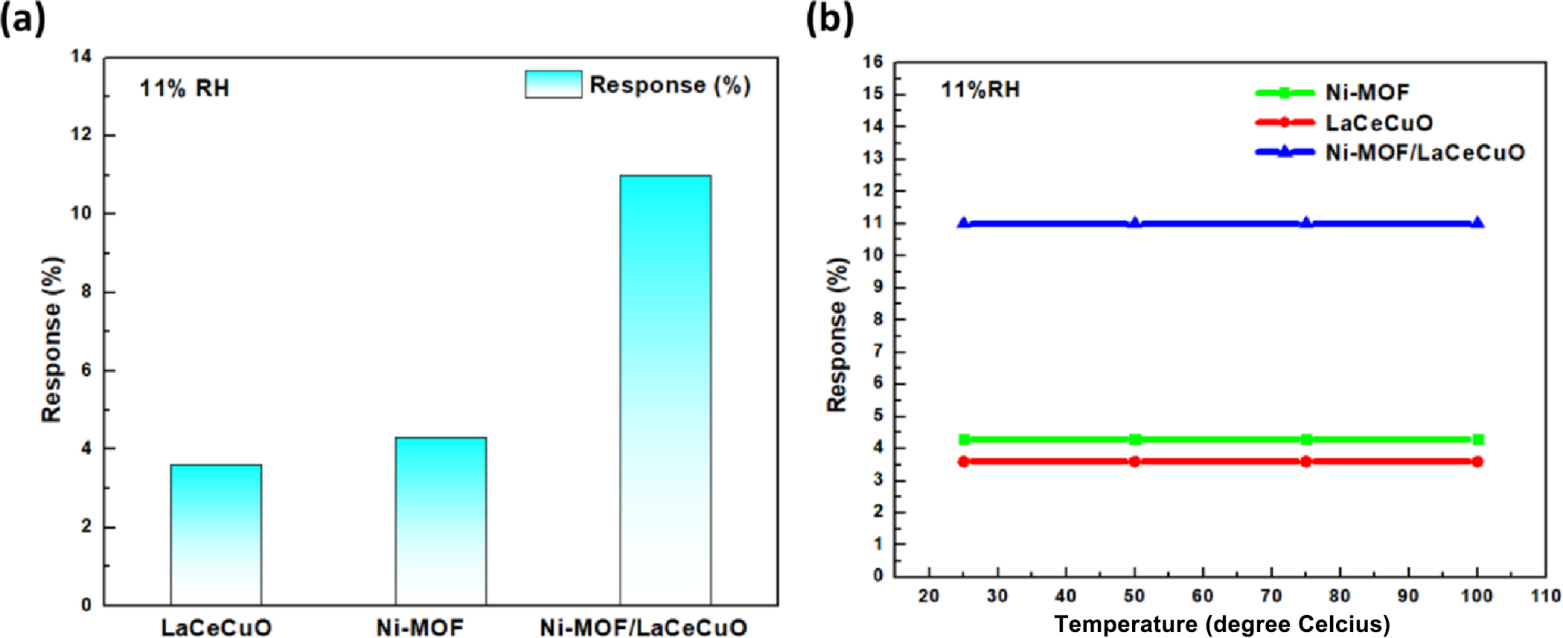
(a) Response of the three sensors (LaCeCuO, Ni-MOF, and LaCeCuO/Ni-MOF) at 11% RH levels. (b) Effect of temperature on sensor response at 11% RH levels.

To investigate the saturation behavior of the proposed CeLaCuO/Ni-BTC sensor, an experimental test was carried out across a humidity range of 11% to 100% RH. As illustrated in Figure 6a, the resistance variation over time with respect to relative humidity indicates that the CeLaCuO/Ni-BTC composite reached full saturation at 74% RH. Furthermore, as shown in Figure 6a, the sensor’s response curves for both increasing and decreasing humidity levels are nearly symmetrical, demonstrating the reversibility of the adsorption and desorption processes, with a maximum humidity hysteresis of <1% observed at 43% RH, Figure S8. The resistance value of the sensor under different RHs was monitored over time, demonstrating three different operational phases: baseline stability (in dry conditions), humidity exposure (with a significant drop in resistance), and recovery (return to baseline upon vacuum application); see Figure 6b. The observed high reduction in resistance with the increase of % RH demonstrates the highly sensitivity of CeLaCuO/Ni-BTC response to water molecules, which enhances charge carrier mobility and alters its electrical conductivity. Hence, we further investigated the sensitivity of the proposed sensor. As shown in Figure 6c and Figure S7, the results demonstrate that the CeLaCuO/Ni-BTC humidity sensor exhibited a high humidity sensitivity of *S* = 1.35/%RH and presented an excellent linear relationship (*R* ^2^ = 0.9868) between 11% RH and 63% RH.

**Figure 6:**
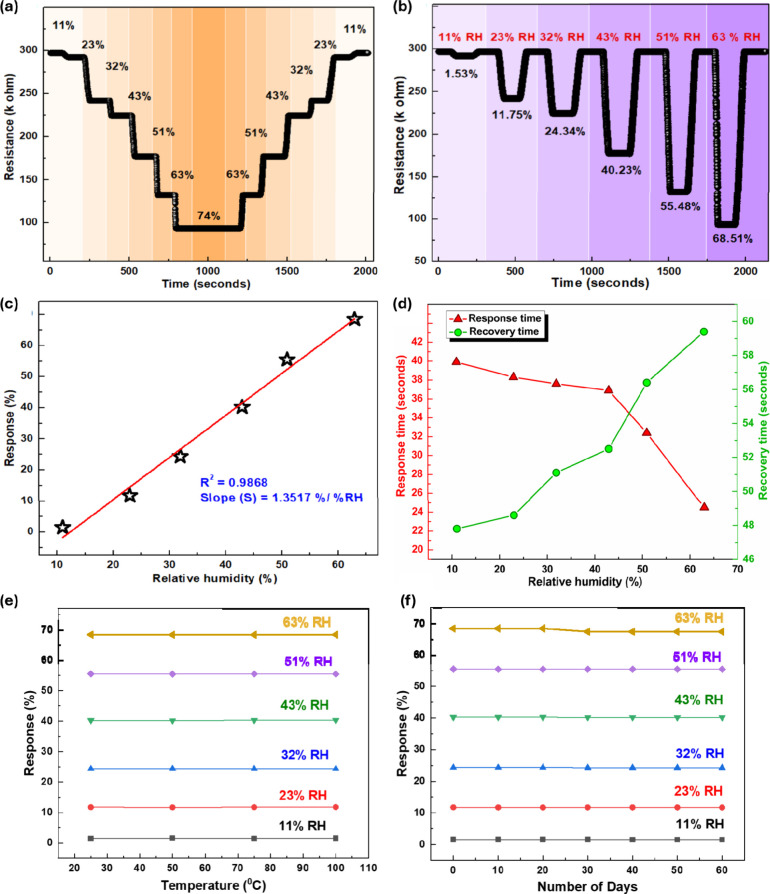
(a) Resistance response curve of the CeLaCuO/Ni-BTC sensor from high to low and then low to high relative humidity (RH). (b) Response–recovery curve of the proposed sensor under gradually increasing and decreasing % RH. (c) Sensitivity and humidity hysteresis analysis. (d) Response and recovery analysis at different levels of RH in the range of 11% RH to 63% RH. (e) Cross-sensitivity analysis with temperature variation. (f) Long-term stability of the CeLaCuO/Ni-BTC sensor at different relative humidities.

Moreover, the response (τ_res_) and recovery (τ_rec_) times of the proposed sensor were evaluated across a relative humidity (RH) range of 11–63%, demonstrating rapid response with τ_res_ ranging from 24.5 to 39.9 s and τ_rec_ ranging from 47.8 to 59.4 s, as shown in Figure 6d and Figure S7a–f.^54^ Notably, the response time decreased with increasing RH, which can be attributed to stronger interactions between water molecules and the sensor surface at higher humidity levels, facilitating faster adsorption and charge transport processes. In contrast, the recovery time exhibits slight variations, likely due to the stronger adsorption of water molecules at elevated RH, which mildly delays the desorption process. This behavior highlights the balance between adsorption kinetics and desorption dynamics in determining overall sensor performance across varying humidity conditions.^55^ Additionally, cross-sensitivity to temperature is a critical factor in evaluating the performance of the proposed humidity sensor. Figure 5e and Figure S9 show the proposed humidity sensor’s cross-sensitivity analysis by measuring the sensitivity fluctuation while varying the temperature across a controlled range of 25–100 °C. The results revealed minimal deviation in sensor response (<0.35%), indicating that the sensor maintains excellent stability under different thermal conditions. This low cross-sensitivity highlights the robustness and reliability of the sensor for practical applications. Moreover, Figure 6f and Figure S9 present the sensitivity values of the proposed sensor over 60 days at various %RH levels, demonstrating its excellent long-term stability (<2.6%).

### 3.5 Humidity-Sensing Mechanism of the CeLaCuO/Ni-BTC Humidity Sensor

The humidity sensing mechanism of the CeLaCuO/Ni-BTC composite involves three key processes: water adsorption, proton conduction, and charge transfer, which combine to influence the properties. When exposed to humid conditions, water molecules from the air are adsorbed onto the surface of the CeLaCuO/Ni-BTC composite.^56^ The interaction is represented in eq 1 in the Supporting Information. On the surface of the Ni-BTC MOF, numerous water molecule assembles into multilayers, as represented in eq 2 in the Supporting Information.^56^ Adsorbed water undergoes self-ionization, leading to the formation of hydronium (H_3_O^+^) and hydroxide (OH^–^) ions^57^ (eq 3 in the Supporting Information). These protonic species enhance charge transport, contributing to proton conduction within the sensing layer (eq 4 in the Supporting Information).^58^ The CeLaCuO oxide contains oxygen vacancies, facilitating interaction with adsorbed water, modifying the electronic properties.^59^ Aliovalent substitution of Ce^4+^ by La^3+^ and Cu^2+^ generates charge-compensating oxygen vacancies (VO··) according to the Kröger–Vink formalism.^60^ Such defect engineering in ceria is well established to enhance adsorption and proton conduction.^61^ The defect chemical equation is listed in the SI. In our composite, the presence of oxygen vacancies is directly supported by multiple experimental signature results discussed above. Particularly, O 1s XPS spectra exhibit a distinct high-binding-energy component (∼531.5–532 eV) corresponding to adsorbed oxygen species at defect sites, in addition to the lattice oxygen peak at ∼529.5 eV.^62^ Ce 3d spectra confirm the coexistence of Ce^3+^ and Ce^4+^, where Ce^3+^ stabilization is a hallmark of oxygen-deficient ceria environments.^63^ Raman analysis shows a downward shift of the F_2_g mode from ∼466 cm^–1^ (stoichiometric CeO_2_) to ∼449 cm^–1^, consistent with lattice distortion induced by oxygen vacancies, and IFFT-processed HRTEM images reveal local lattice distortions at the oxide/MOF interface, further indicating defect incorporation. Together, these results confirm that abundant oxygen vacancies are present in the CeLaCuO/Ni-BTC phase and play a key role in enabling fast and reversible water adsorption during humidity sensing.

Furthermore, eq 5 in the Supporting Information represents the reaction responsible for modulating surface conductivity and altering electrical resistance. The presence of water affects the hole concentration in the sensing material.^64^ Water molecules interact with copper ions in the composite, contributing to charge transfer and resistance changes. The formation of hydroxide ions (OH^–^) and hole carriers (h^+^) influences the overall conductivity (eq 6).

The linear relationship in Figure 6 reflects the “overall effective response” of the sensor within the tested humidity range, where the combined effect of both physisorption and chemisorption sites leads to a near-linear dependence on humidity concentration. Although a multiadsorption mechanism was observed at lower and moderate humidity levels, the dominant contribution arises from physisorbed water molecules forming the first and second adsorption layers. These layers provide continuous proton conduction through the Grotthuss mechanism, resulting in a quasi-linear response. At higher humidity levels, additional chemisorption and capillary condensation effects occur, but within the tested range, these contributions remain proportionally balanced, thus maintaining the observed linearity. The adsorbed water molecules desorb when the humidity source is removed, restoring the sensor to its previous electrical state^65,66^ (Figure 7a) (eq 7). To further clarify the sensing mechanism, we distinguish between electronic conduction, which is typically temperature-sensitive in oxide sensors, and protonic conduction, which dominates in our composite and explains the observed thermal robustness. In conventional oxide-based humidity sensors (e.g., ZnO, SnO_2_, WO_3_), the sensing response is dominated by electronic conduction through the oxide lattice, where surface-adsorbed oxygen species capture or release electrons from the conduction band.^67–69^ The resulting carrier density and mobility follow an Arrhenius-type relationship, making the sensitivity strongly temperature dependent. By contrast, in our CeLaCuO/Ni-BTC sensor, the conduction pathway is governed by protonic transport through adsorbed water networks, as evidenced by the nearly temperature-invariant sensitivity (<0.35% deviation across 25–100 °C). This protonic conduction occurs via the Grotthuss mechanism,^69^ in which protons (H^+^/H_3_O^+^) hop between adjacent water molecules along hydrogen-bonded chains confined within the MOF pores and stabilized at oxygen vacancies in CeLaCuO/Ni-BTC.^70–73^ Oxygen vacancies, confirmed in our material by O 1s and 3d atoms of Ce as well as Raman shifts, provide energetically favorable adsorption sites that anchor water molecules and facilitate dissociative adsorption. The Ni-BTC framework further supports the formation of continuous hydrogen-bonded networks due to its hydrophilic carboxylate groups and thermally stable porosity up to 100 °C. Consequently, while electronic conduction would be expected to show strong thermal activation, the dominant humidity-controlled proton-hopping pathway remains largely unaffected by temperature variations, explaining the robustness of our sensor.

**Figure 7:**
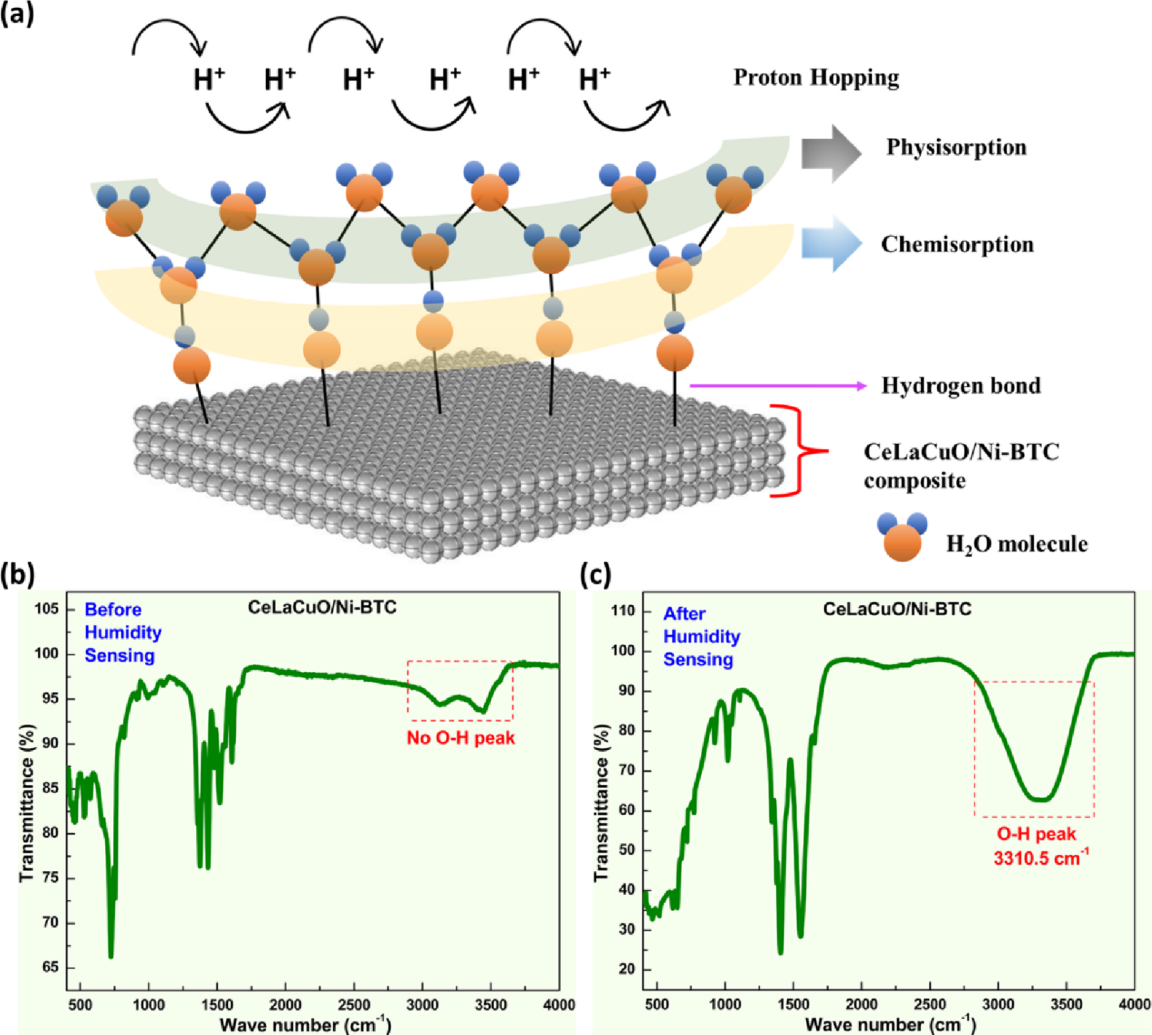
(a) Humidity sensing mechanism for the CeLaCuO/Ni-BTC composite and FT-IR spectra for the CeLaCuO/Ni-BTC composite (b) before and (c) after the humidity sensing test.

The FT-IR spectra before (Figure 7b) and after (Figure 7c) humidity sensing show significant changes in the O–H stretching region (3200–3600 cm^–1^). After humidity exposure, the peak broadens and intensifies, indicating increased hydrogen bonding due to water absorption. A redshift occurs due to stronger interactions, while the H–O–H bending vibration (∼1600 cm^1^) also increases.

Water adsorption occurs through different layers of the composite material. In any case, the general reaction reflecting the phenomenon is given below:

$$M_{2}O_{x}+xH_{2}O\to 2M(\mathrm{OH}{)}_{x}$$

As per the reaction, it is anticipated that depending on the extent of hydration (number of layers of materials participating), M­(OH)*_x_* species will coexist alongside the M_2_O_*x*_ ones (coexistence of metal oxide and metal hydroxide). This is reflected in the FTIR spectra by the profound increase of the O–H band at both stretching (≈3000 cm^–1^) (stretching vibration of O–H) and bending vibration regions (≈1500 cm^–1^). Adsorption of water is also anticipated to induce changes in the M–O fingerprint peaks (400–700 cm^–1^); in the spectra presented in Figure 6a,b, the peak at 750 cm^–1^ dramatically decreases in intensity as the metal sites participate in the interaction with the water molecule to form M–OH bonds, ultimately altering the original metal oxide lattice structure near the surface. These spectral variations confirm the material’s responsiveness to humidity and highlight its potential for humidity sensing applications.

Finally, a comparative analysis of various MOF-based humidity sensors is presented in Figure 8. The proposed sensor demonstrates a good performance relative to previously reported devices. It exhibits high sensitivity (1.35/%RH), excellent long-term stability (60 days), low hysteresis (0.465%), and excellent linearity (0.9868) across the tested humidity range (11% RH to 63% RH). Additionally, the sensor shows a low cross-sensitivity to temperature, ultrafast response (∼24.5 s), and recovery (∼47.8 s) times, which are critical for real-time monitoring. These combined features highlight the sensor’s potential for practical applications in advanced humidity sensing systems.

**Figure 8:**
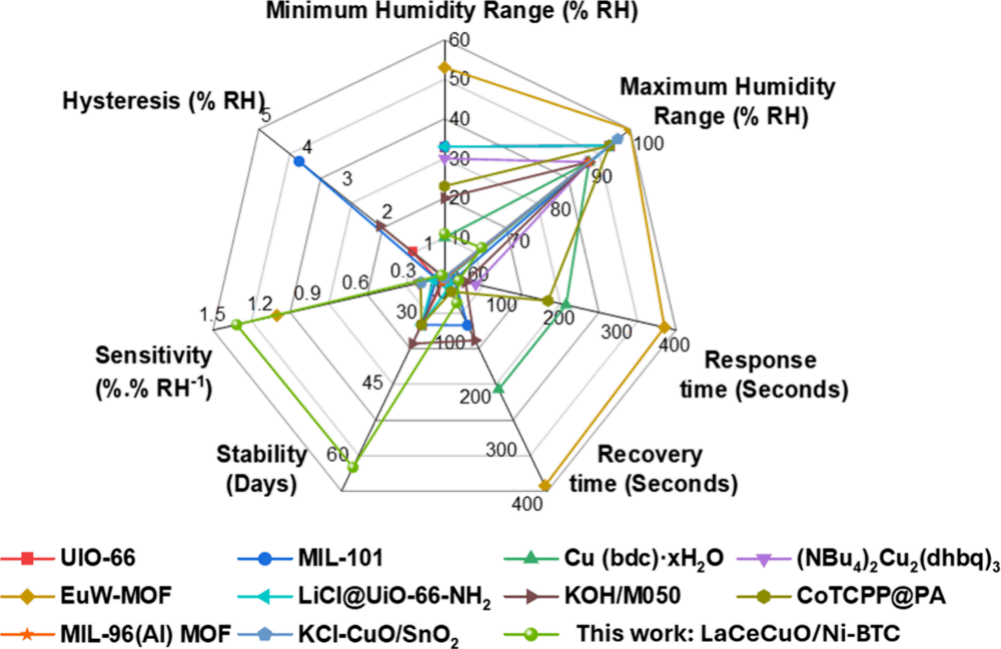
Spider chart showing the performance metrics of different MOF-based humidity sensors.^59,74–80,83,84^

### 3.6 Microclimate System with Varying Humidity Levels for a Real-Life Sensor Evaluation: The Case Study of the *Fragaria ananassa* Plant

To assess the real-world applicability of the CeLaCuO/Ni-BTC humidity sensor, a controlled microclimate chamber was designed to simulate varying humidity environments and evaluate plant response. *Fragaria ananassa* (strawberry), a commercially significant and climatically sensitive crop, was chosen as a model plant.^81,82^ This crop is particularly valued due to its shallow root system, high water demand, and sensitivity to environmental stress.^81^ As climate variability increases and water resources become scarcer, optimizing humidity conditions for crops such as strawberries is essential to ensure food security, reduce yield loss, and promote sustainable cultivation practices.

An enclosed microclimate chamber was developed utilizing saturated salt solutions to maintain specific humidity levels to determine the ideal humidity level for the *Fragaria ananassa* plant in a controlled setting (Figure S10). The saturated solutions of sodium nitrite (NaNO_2_), magnesium nitrate (Mg­(NO_3_)_2_), and potassium carbonate (K_2_CO_3_) were used to maintain humidity levels at 63%RH, 51%RH, and 43%RH, respectively. The 13 L chamber attaining equilibrium with the specific humidity levels took almost an hour (30 min). A standard reference sensor and a CeLaCuO/Ni-BTC sensor were connected inside the chamber for comparative measurements. Over 3 days, the *Fragaria ananassa* plant was kept inside a chamber; changes in its leaves were observed to measure the plant’s tolerance to the humidity. Significant physiological responses were revealed after a three day plant observation and under varying relative humidity (RH) conditions. At low humidity conditions (51% RH and 43% RH), the *Fragaria ananassa* plant experienced increased transpiration rate, leading to excessive water loss from leaves, which resulted in wilting, leaf curling, and stunted growth due to water stress (Figure 9c, d, e). The stomata remained open, resulting in longer, accelerating moisture loss and increasing the risk of dehydration, affecting the overall quality of the plant. At 63% RH (NaNO_2_), plants exhibited optimal transpiration, allowing efficient water and nutrient uptake, resulting in healthy leaf morphology with minimal stress. Figure 9c clearly shows wilting of the *Fragaria ananassa* plant at 43% RH, while at 63% RH, it was pretty healthy (Figure S11).

**Figure 9:**
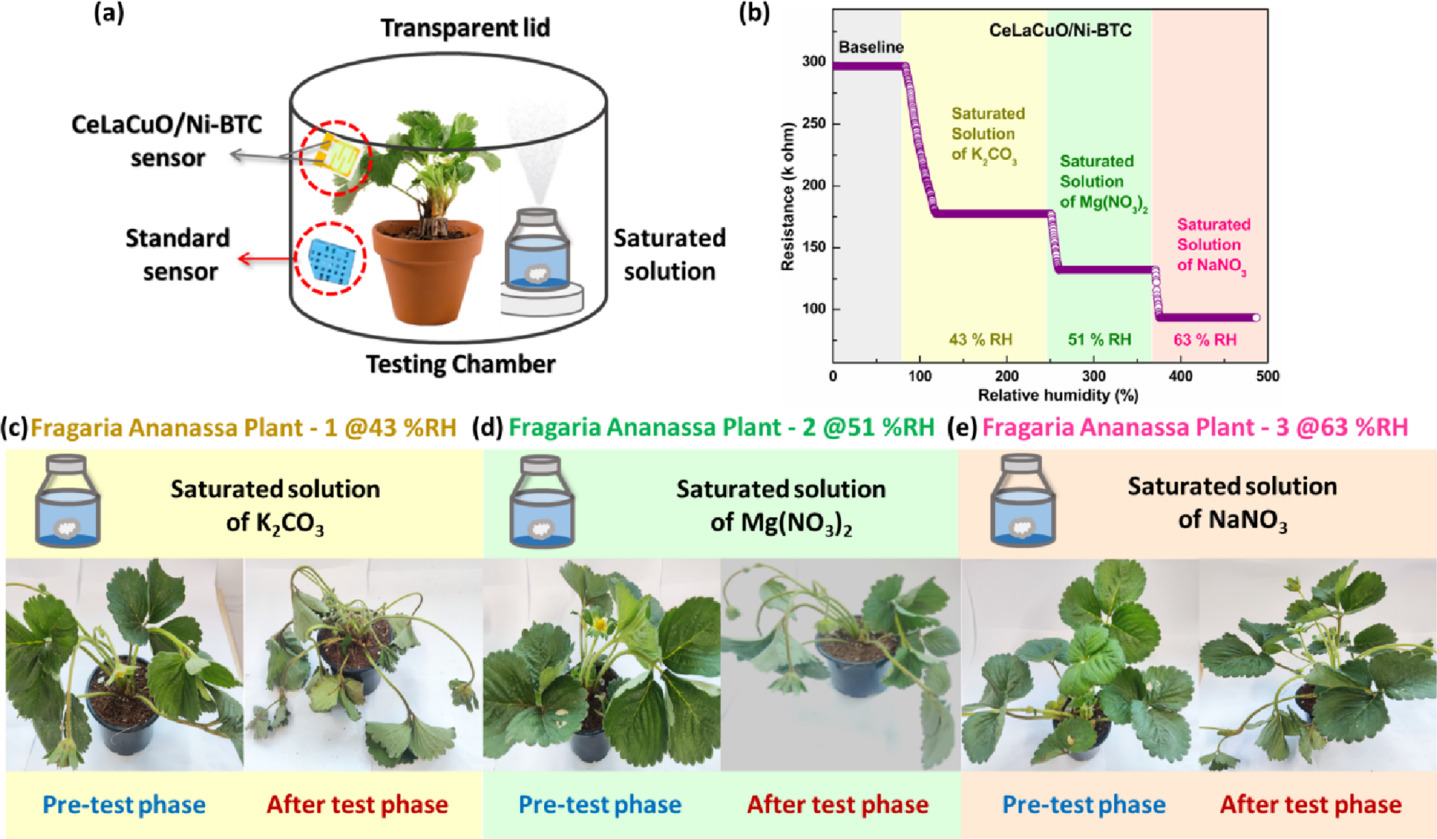
(a) Testing chamber with the *Fragaria ananassa* plant under different relative humidity levels. (b) Response of the CeLaCuO/Ni-BTC sensor in the testing chamber at three different saturated solutions. The *Fragaria ananassa* plant was tested at (c) 43%RH, (d) 51%RH, and (e) 63%RH.

## Conclusions

In this study, the CeLaCuO/Ni-BTC composite was successfully synthesized and characterized; it demonstrated that the oxygen vacancy rich structure leads to a high-performance humidity sensor. The incorporation of CeLaCuO into the Ni-BTC MOF resulted in a hierarchical hybrid material with enhanced water adsorption properties owing to the synergistic effects of oxygen vacancies, high porosity, and metal–organic framework stability. Structural characterization via PXRD, Raman spectroscopy, and HRTEM confirmed the successful integration of the CeLaCuO phase into the Ni-BTC MOF without phase separation. At the same time, elemental mapping validated the uniform distribution of Ce, La, Cu, Ni, and O elements throughout the composite. The humidity sensing performance of the CeLaCuO/Ni-BTC composite was evaluated using resistive measurements, demonstrating a fast response time of 24.5 s and a recovery time of 47.8 s, high sensitivity (slope = 1.35/%RH), good linearity (*R* ^2^ = 0.9868) between 11% RH and 63% RH, low cross-sensitivity to temperature (25–100 °C, <0.35%), and long-term stability over 60 days (<2.6%). The humidity sensing mechanism was elucidated, revealing the crucial roles of oxygen vacancies, proton conduction, and charge transfer in modulating the sensor response. The combination of MOF porosity and oxide redox chemistry in this work establishes a new class of hybrid material for reliable and scalable humidity sensing technologies. Additionally, real-world validation using a microclimate chamber and plant health monitoring (*Fragaria ananassa*) highlights the sensor’s potential for smart greenhouses, agricultural, and environmental applications.

## Supplementary Material



## Notes

XPS spectra of Ni-BTC; IFFT-processed lattice images of CeLaCuO/Ni-BTC; HR-TEM, HAADF-STEM, and EDS elemental maps of CeLaCuO and Ni-BTC; schematic of the humidity sensing setup and sensor fabrication; response curves of CeLaCuO, Ni-MOF, and CeLaCuO/Ni-MOF sensors at various relative humidities; hysteresis, repeatability, temperature, and long-term stability plots; photographs and physiological observations of *Fragaria ananassa* plants under different humidity levels; proposed humidity-sensing reaction scheme and defect-chemistry equations (PDF)
