# Supplementary material for: Highly Sensitive and Stable CeLaCuO/Ni-BTC MOF-Based Humidity Sensor for Plant Monitoring
Source: ACS Appl Mater Interfaces. 2025 Dec 6;17(50):67823–36. doi: 10.1021/acsami.5c16966 (PMC12723648; doi:10.1021/acsami.5c16966)
Supplement: Supplementary file 1 [file am5c16966_si_001.pdf]

# Supplementary material

# Highly Sensitive and Stable CeLaCuO/Ni-BTC MOF-Based Humidity Sensor for Plant Monitoring

Jolina Rodrigues[1#] , Karthik Chimatahalli Santhakumar[1,2#], Hamid Nawaz[1], Swati Singh[1,2], Smruti Medha Mishra[3], Dalaver Hussain Anjum[2,3], Kyriaki Polychronopoulou[1,2*] and Nouha Alcheikh[1, *]

[1]Department of Mechanical and Nuclear Engineering, Khalifa University of Science and Technology, Main Campus, Abu Dhabi, P.O. Box 127788, UAE

[2]Center for Catalysis and Separations (CeCaS), Khalifa University of Science and Technology, Main Campus, Abu Dhabi, P.O. Box 127788, UAE

[3]Department of Physics, Khalifa University of Science and Technology, 127788, Abu Dhabi, United Arab Emirates

*# Equally contributed as first author*

**Corresponding Email:* kyriaki.polychrono@ku.ac.ae *and* nouha.alcheikh@ku.ac.ae

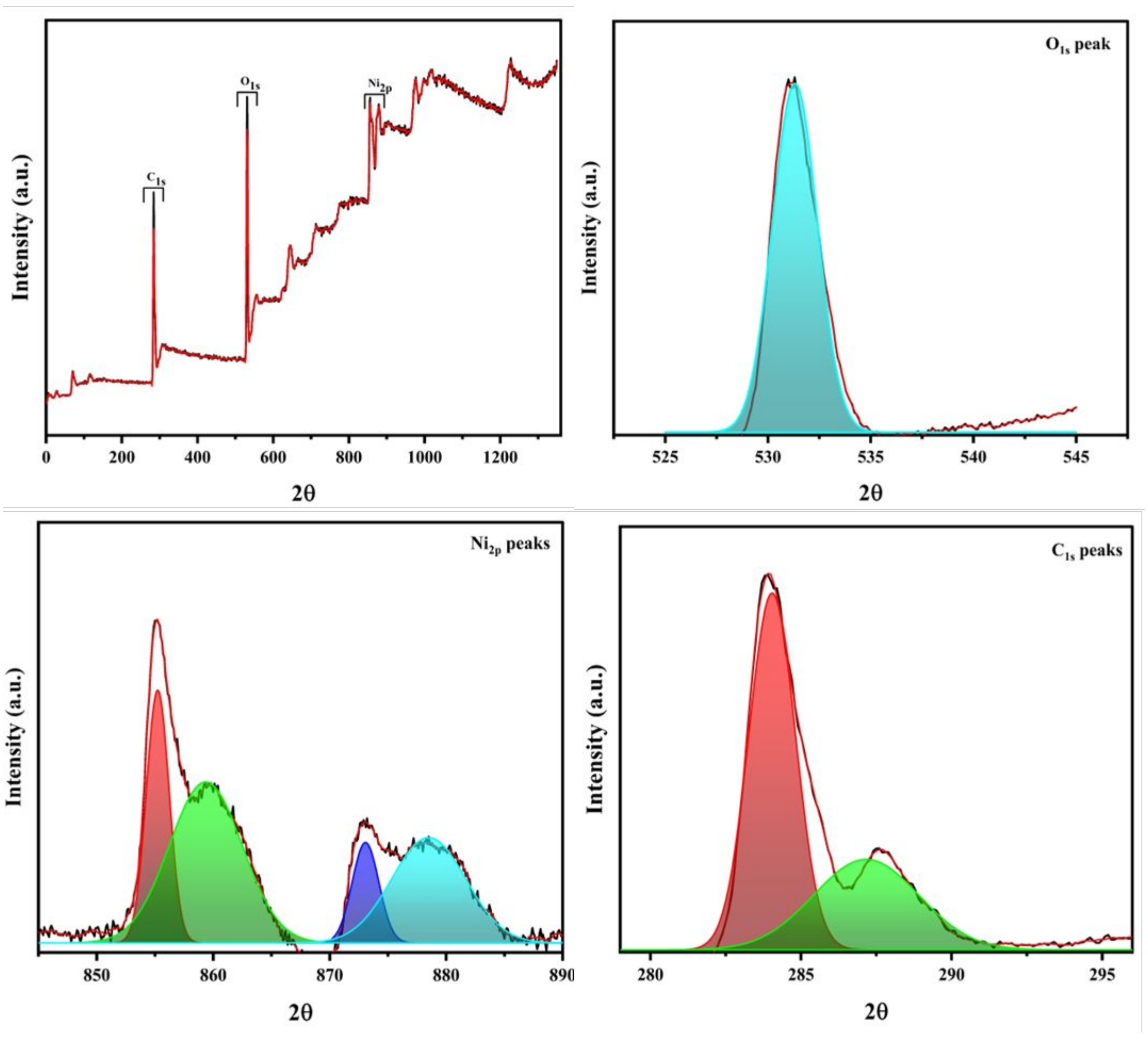


**Figure S1.** X-ray photoelectron spectroscopy (XPS) spectra of Ni-BTC MOF: (top-left) survey scan, (top-right) high-resolution O 1s, (bottom-left) high-resolution Ni 2p, and (bottom-right) high-resolution C 1s spectra

The survey spectrum confirms the presence of C 1s, O 1s, and Ni 2p, corresponding to the organic linker and metal center in the Ni-BTC framework. The O 1s high-resolution spectrum shows a dominant peak centered around ~530.2 eV, attributed to lattice oxygen bonded to Ni, indicating a stable coordination environment. The Ni 2p spectrum exhibits distinct spin–orbit doublets at ~855.2 eV and ~872.8 eV for Ni $2p_3/_2$ and Ni $2p_1/_2$, respectively, with accompanying satellite peaks, confirming the presence of $Ni^{2+}$ in the framework. The C 1s

spectrum presents a major peak at ~284.6 eV, corresponding to adventitious carbon, and a secondary component at ~288.7 eV, attributed to O–C=O bonds from the carboxylate groups of the BTC linker.

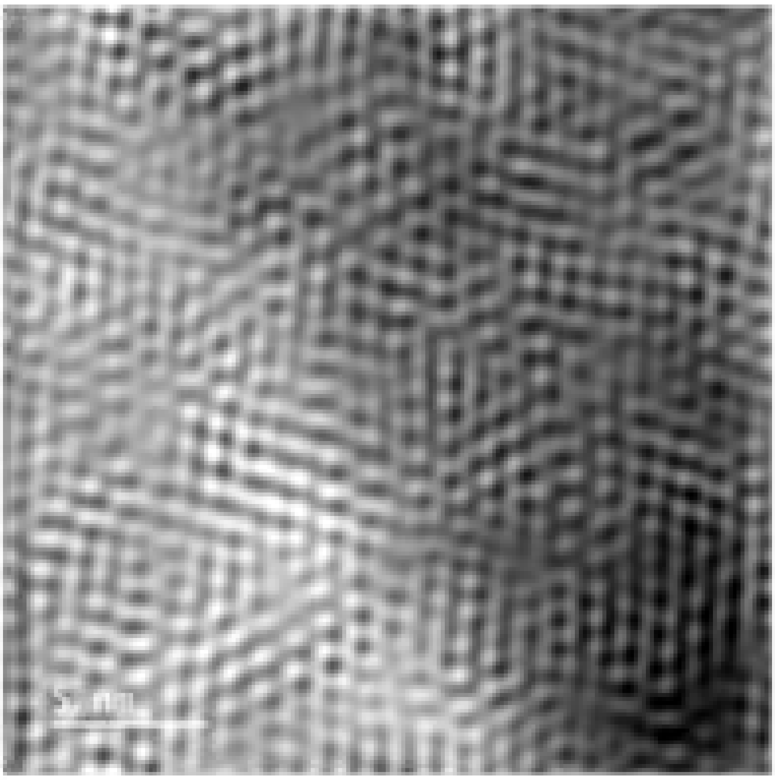

**Figure S2.** The inverse fast Fourier transform (IFFT) processing enhances lattice visibility, highlighting the interplanar spacing and structural integration between CeLaCuO and Ni-BTC MOF

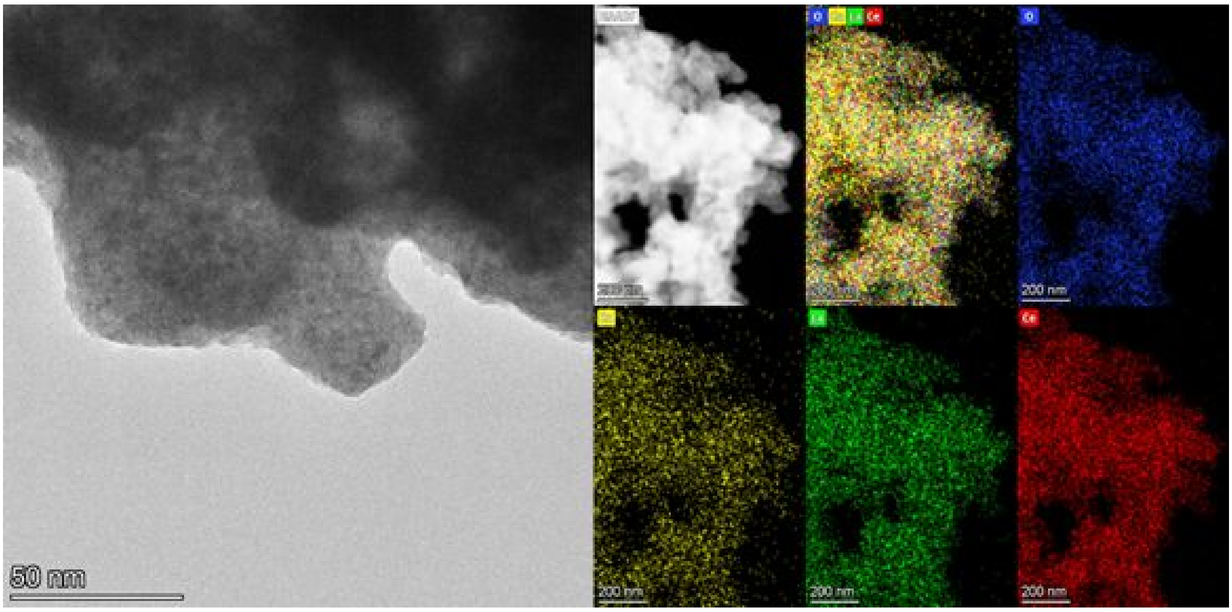


**Figure S3.** High-resolution transmission electron microscopy (HR-TEM), high-angle annular dark-field scanning transmission electron microscopy (HAADF-STEM), and energy-dispersive X-ray spectroscopy (EDS) elemental mapping of CeLaCuO composite

The HR-TEM image confirms the porous nanostructure of the composite, while the HAADF-STEM image highlights regions of high atomic number contrast, attributable to the Ce, La, and Cu-rich domains. Elemental mapping reveals the uniform distribution of Ce, La, Cu, and O throughout the material, confirming successful ternary metal oxide of CeLaCuO.

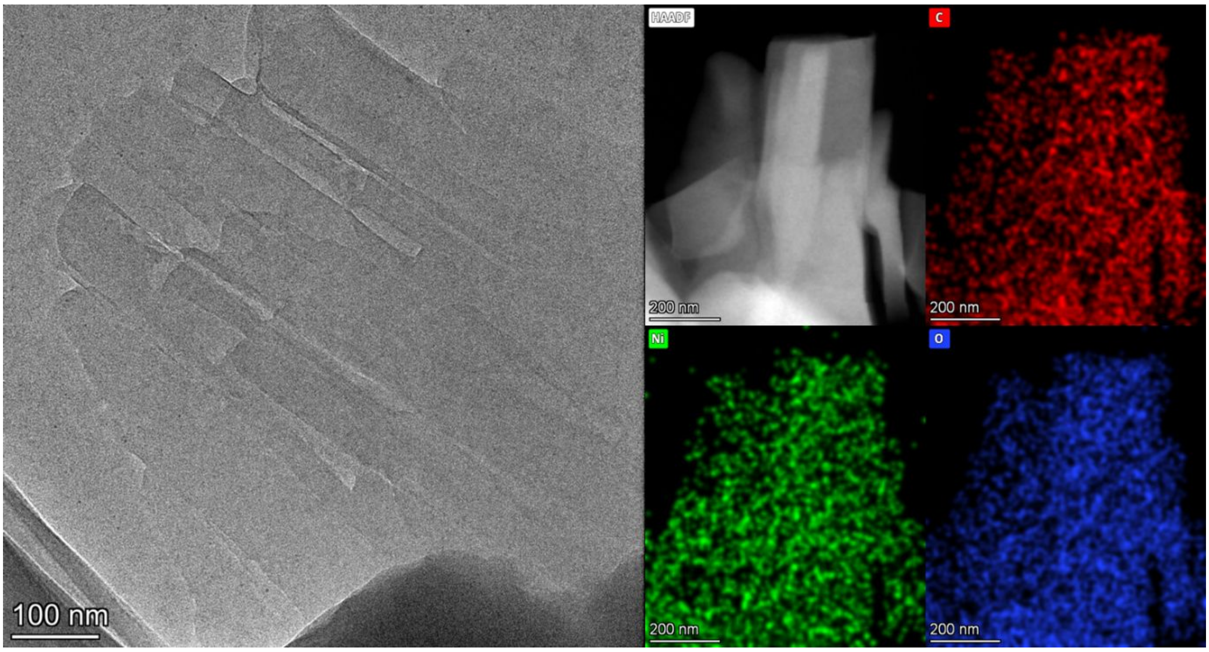


**Figure S4.** HR-TEM, HAADF-STEM, and EDS elemental mapping of pristine Ni-BTC MOF

The HR-TEM micrograph reveals well-defined lamellar morphology characteristic of Ni-BTC, while HAADF-STEM imaging and elemental maps for Ni, C, and O demonstrate uniform distribution of framework constituents. The absence of Ce, La, and Cu validates the pristine nature of the MOF prior to composite formation. The preserved crystallinity and structural uniformity serve as an effective platform for the subsequent incorporation of CeLaCuO oxide.

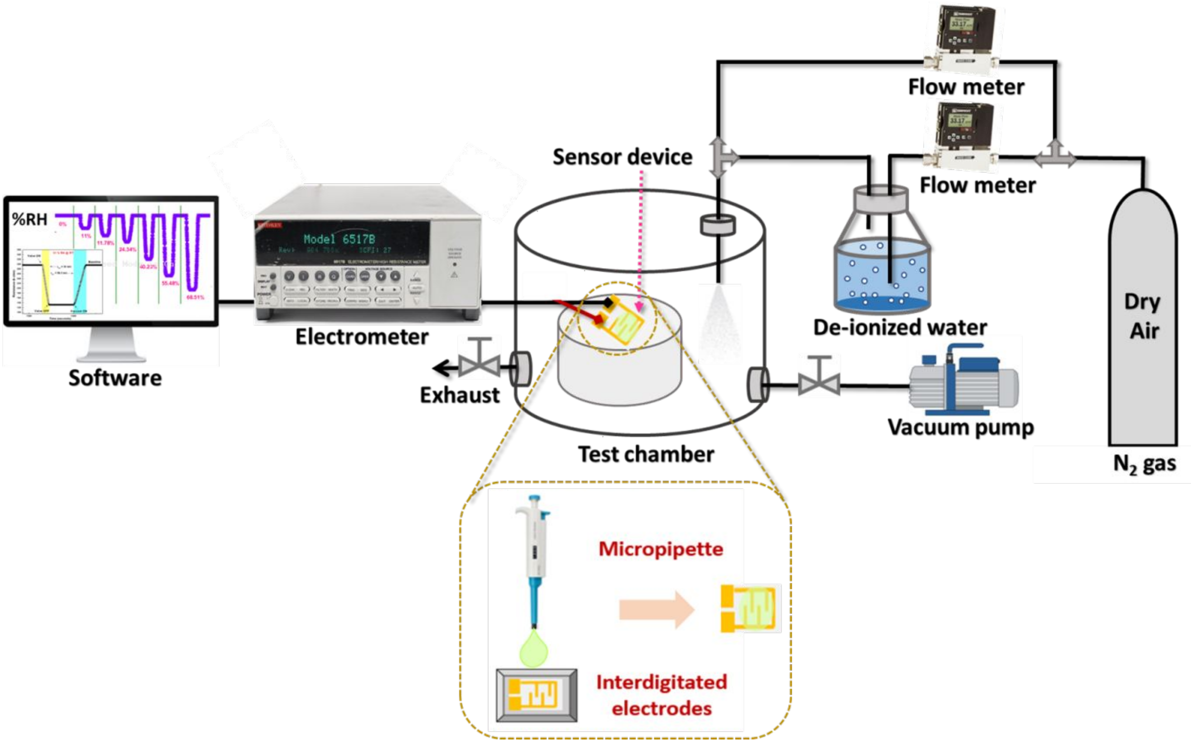


**Figure S5.** Schematic representation of Humidity sensing setup, (Inset) Fabrication of sensor device via drop casting method

A sealed chamber, source meter, flow meter, vacuum pump, nitrogen gas cylinder, bubbler (humidifier), and data acquiring software are essential parts of the humidity sensor setup illustrated above. To ensure a regulated testing environment, the chamber is first evacuated using a vacuum pump to remove ambient air. After the vacuum is reached, the cylinder valve is opened to introduce nitrogen gas, which flows through a bubbler containing distilled water. According to the flow speed and chamber volume, this process generates nitrogen gas that is saturated with water vapor, resulting in a specific relative humidity. The chamber containing the sensor is filled with the humidified gas. The source meter records the electrical response of the sensor to varying humidity levels in real time, and software is then used to evaluate the

data. To assess sensor performance attributes including sensitivity, reaction time, and stability, experiments are carried out at various relative humidity levels.

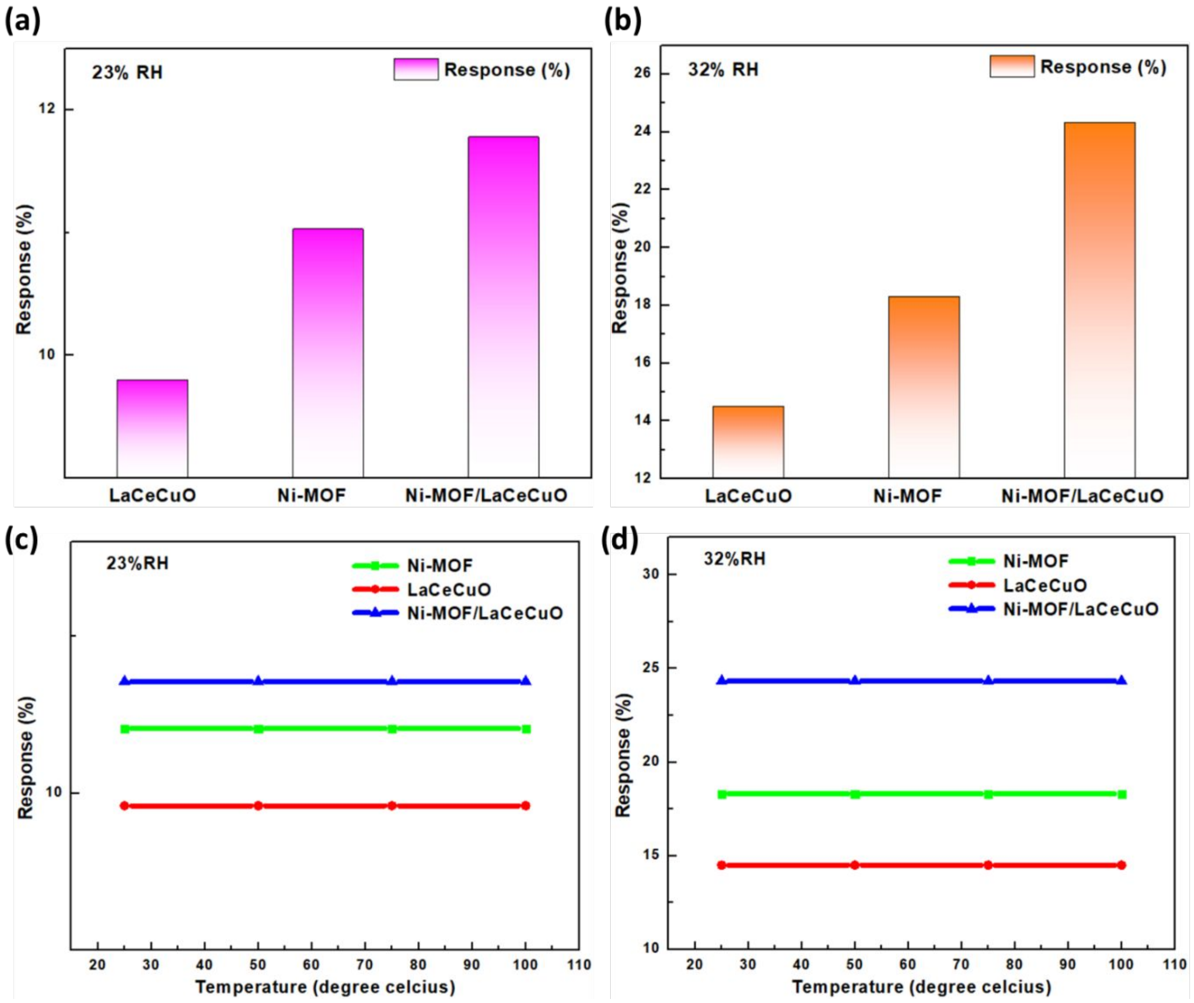


**Figure S6.** Response of the three sensors (LaCeCuO, Ni-MOF, and LaCeCuO/ Ni-MOF) at (a) 23 % RH levels, (b) 32 % RH levels, Effect of temperature on sensor response at (c) 23 % RH levels, (d) 32 % RH levels

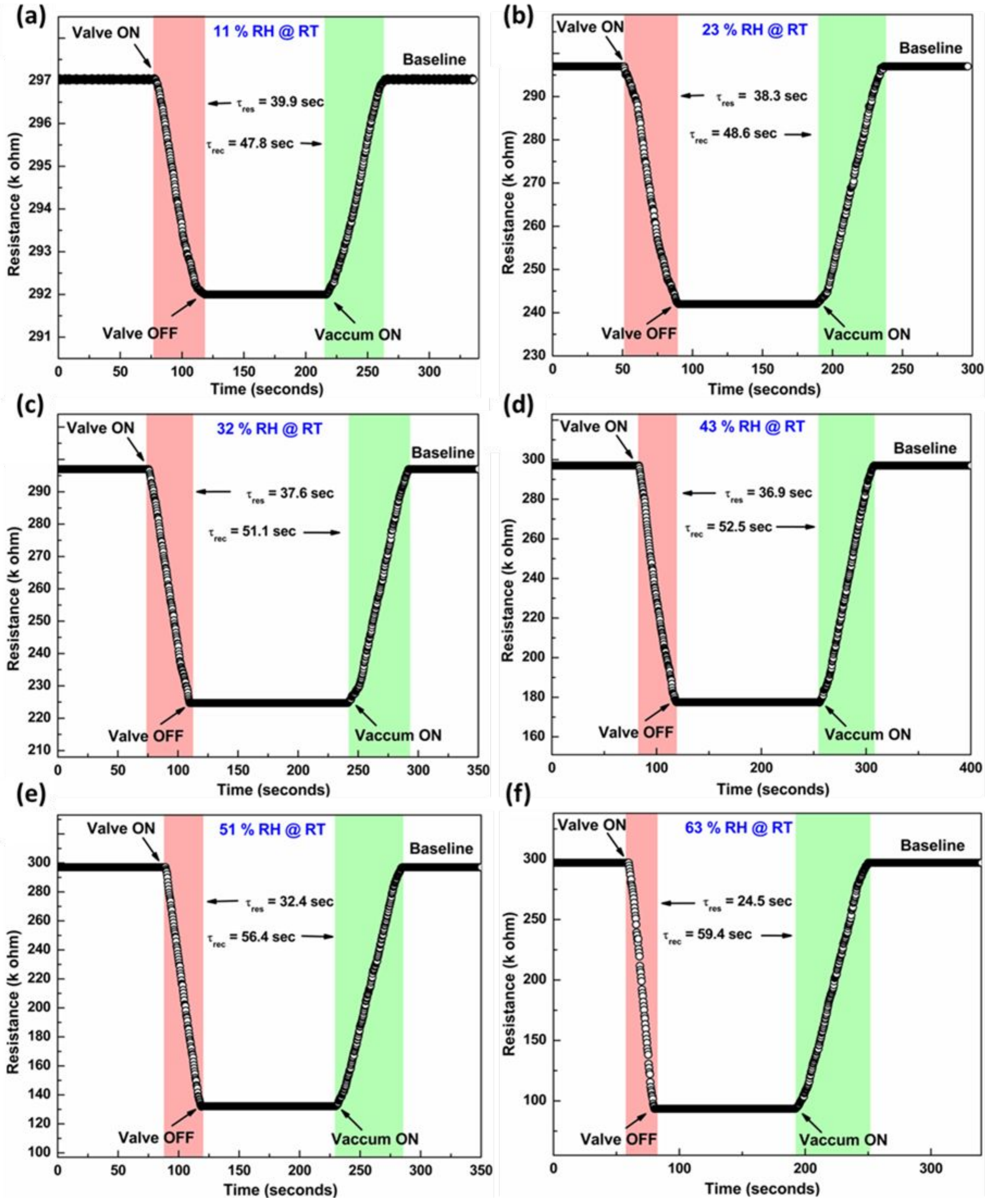


Figure S7. CeLaCuO/Ni-BTC composite response at (a) 11% RH, (b) 23 % RH, (c) 32% RH, (d) 43% RH, (e) 51% RH and (f) 63% RH

The response time ($\tau_{res}$) observed for 11% RH, 23% RH, 32% RH, 43% RH, 51% RH, 63% RH was 39.9 seconds, 38.3 seconds, 37.6 seconds, 36.9 seconds, 32.4 seconds, 24.5 seconds, respectively. The recovery time ($\tau_{rec}$) observed for 11% RH, 23% RH, 32% RH, 43% RH, 51% RH, 63% RH was 47.8 seconds, 48.6 seconds, 51.1 seconds, 52.5 seconds, 56.4 seconds, 59.4 seconds, respectively (**Figure S7 a-f**). The trend of decreasing response time with increasing RH indicates enhanced water molecule interaction at higher humidity levels, leading to quicker resistance changes. However, the recovery time shows slight variations, suggesting stronger water molecule adhesion at elevated RH, which slightly prolongs desorption.

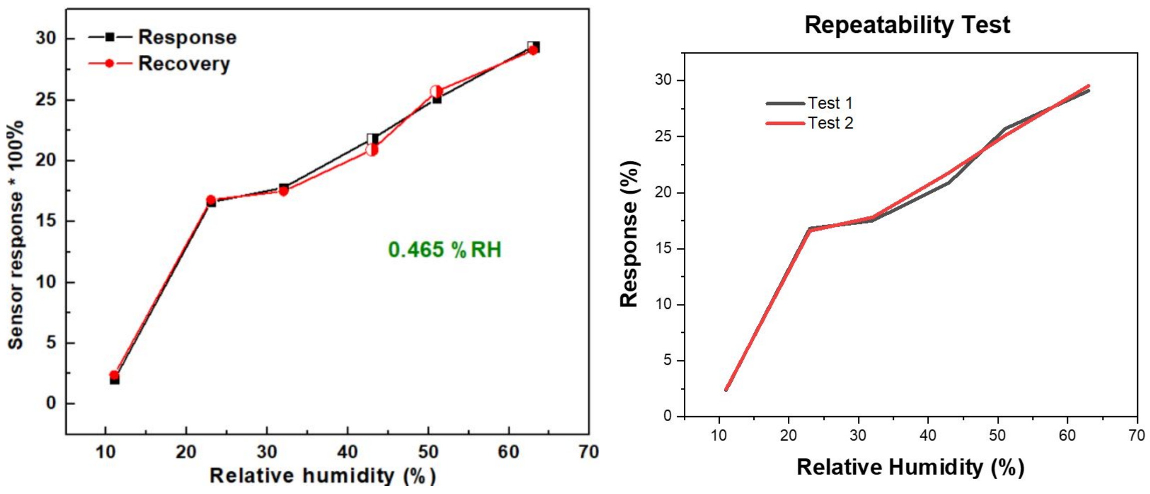


**Figure S8.** Hysteresis and plot for LaCeCuO/Ni-BTC composite along with repeatability test.

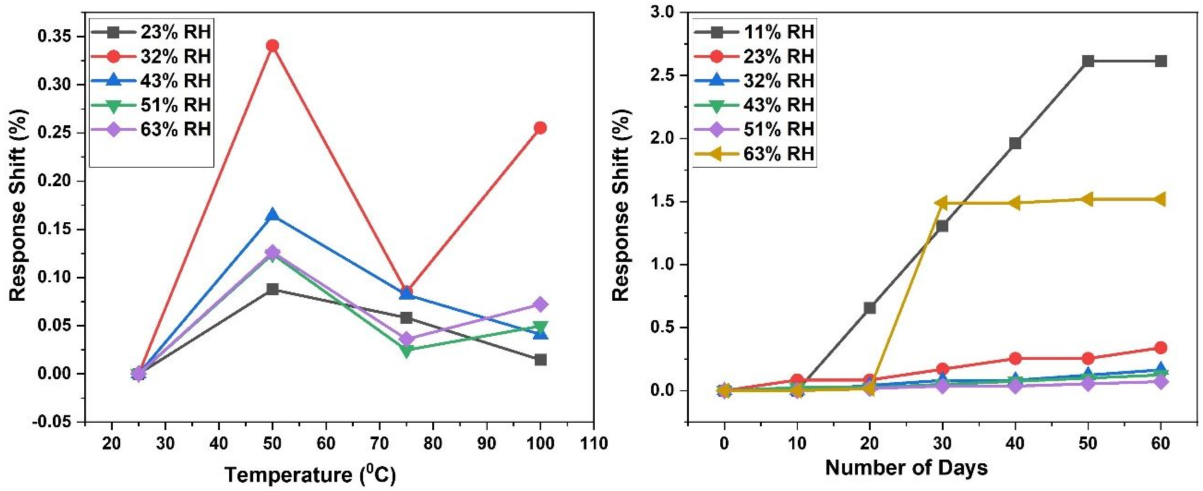


**Figure S9.** Sensor response deviation with temperature (<0.35%) and over 60 days (<2.6%).

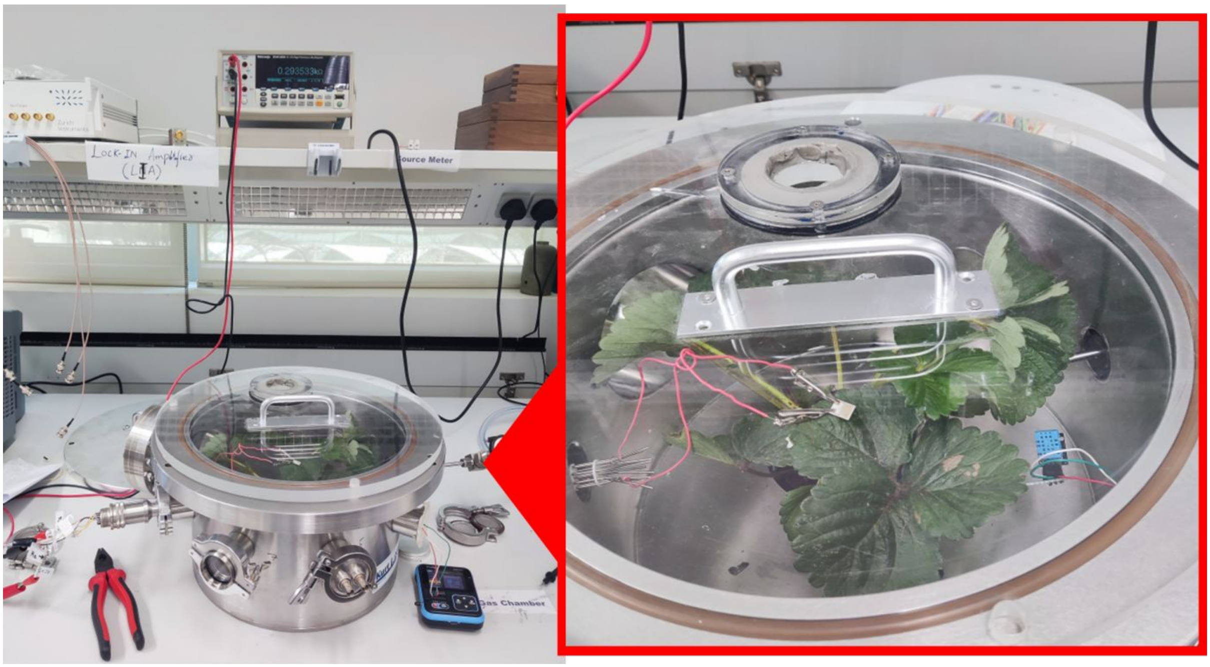

**Figure S10.** Photo taken during humidity sensing experiments were with *Fragaria Ananassa* plant

Photos taken during humidity sensing experimental work were carried out with *Fragaria Ananassa plant.* The standard sensor along with CeLaCuO/Ni-BTC sensor were placed inside

the sealed chamber and connections were made with source meter. Micro climate system was generated inside the chamber using saturated solution placed inside chamber.

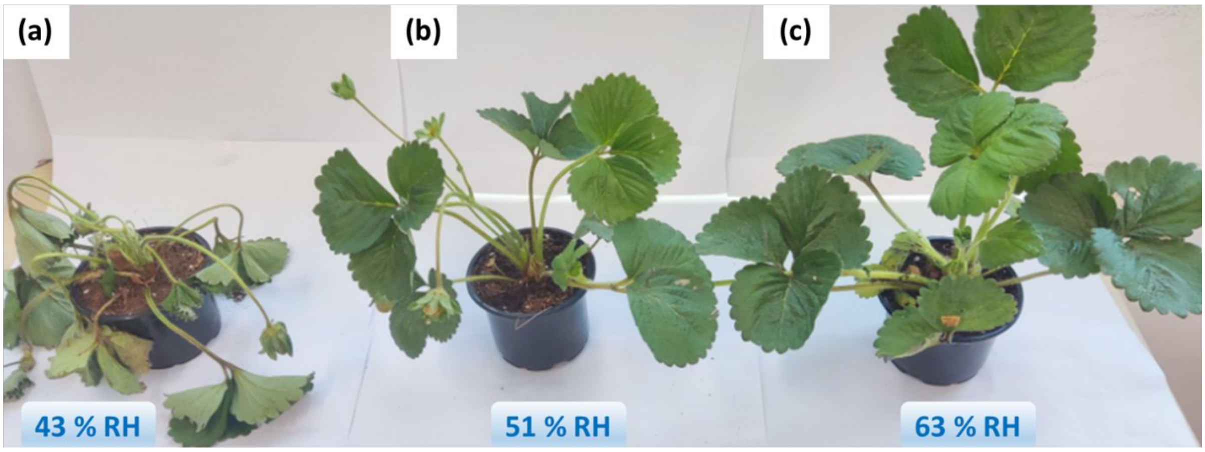


**Figure S11.** Physiological changes in Fragaria Ananassa plant after 3 days at 3 different humidity levels (a) 43 %RH, (b) 51 %RH and (c) 63 %RH

At 63% RH ($NaNO_2$), plants exhibited optimal transpiration, allowing efficient water and nutrient uptake, resulting in healthy leaf morphology with minimal stress. At 63% RH, *Fragaria Ananassa plant* was quite healthy. While at low humidity (43 %RH), *Fragaria Ananassa plant* was under extreme stress. In low humidity conditions (51% RH and 43 % RH), *Fragaria Ananassa plants* experienced increased transpiration, leading to excessive water loss from leaves. Which resulted in wilting, leaf curling, and stunted growth due to water stress.

**Chemical reaction occurring during humidity sensing for CeLaCuO/Ni-BTC composite**

$H_2O_{(gas)} \rightarrow H_2O_{(adsorbed)}$ ……………………………………………………..Equation-1

$nH_2O_{(gas)}$+CeLaCuO/Ni-BTC composite→$(H_2O)_n$ -CeLaCuO/Ni-BTC composite...Equation-2

$2H_2O \rightleftharpoons H_3O^+ + OH^-$ ……………………………………………………..........Equation-3

$H_3O^+ + H_2 O \rightarrow H_2O + H_3O^+$……………………………………………….......…Equation-4

$H_2O + VO^{\cdot\cdot} + O^x \rightarrow 2OH^{\cdot}$ ………………………………………………..........Equation-5

$H_2O + Cu^{2+} \rightarrow Cu^+ + OH^{\cdot} + h^+$…………………………………………………...Equation-6

$(H_2O)_n \rightarrow H_2O_{(gas)}$ ……………………………………………………………Equation-7

**Defect chemistry:**

$La^{3+}$ on a $Ce^{4+}$ site creates oxygen vacancies:

2La'Ce + VO·· +3OO× ↔ La'Ce+ Oox (Charged balance via VO··)

$Cu^{2+}$ on a $Ce^{4+}$ site similarly promotes VO··

Water–vacancy interaction already reported in our SI as

Eq. 5: H2O + VO·· + Ox → 2OH·
